# An interprofessional cohort analysis of student interest in medical ethics education: a survey-based quantitative study

**DOI:** 10.1186/s12910-020-00468-4

**Published:** 2020-04-08

**Authors:** Mikalyn T. DeFoor, Yunmi Chung, Julie K. Zadinsky, Jeffrey Dowling, Richard W. Sams

**Affiliations:** 1 School of Medicine, Medical College of Georgia at Augusta University, Augusta, GA 30909 USA; 2 Institute of Public and Preventative Health, Augusta University, Augusta, GA 30909 USA; 3 Augusta University College of Nursing, Augusta, GA 30909 USA; 4 Augusta University Rehabilitation, Augusta, GA 30909 USA; 5 Center for Bioethics and Health Policy, Medical College of Georgia at Augusta University, Augusta, GA 30909 USA; 6 Department of Family Medicine, Medical College of Georgia at Augusta University, Augusta, GA 30909 USA

**Keywords:** Interprofessional education (IPE), Medical ethics, Healthcare ethics, Medical education, Curriculum

## Abstract

**Background:**

There is continued need for enhanced medical ethics education across the United States. In an effort to guide medical ethics education reform, we report the first interprofessional survey of a cohort of graduate medical, nursing and allied health professional students that examined perceived student need for more formalized medical ethics education and assessed preferences for teaching methods in a graduate level medical ethics curriculum.

**Methods:**

In January 2018, following the successful implementation of a peer-led, grassroots medical ethics curriculum, student leaders under faculty guidance conducted a cross-sectional survey with 562 of 1357 responses received (41% overall response rate) among students enrolled in the School of Medicine, College of Nursing, Doctor of Physical Therapy and BS/(D) MD Professional Scholars programs at The Medical College of Georgia at Augusta University. An in person or web-based questionnaire was designed to measure perceived need for a more in-depth medical ethics curriculum.

**Results:**

The majority of respondents were female (333, 59.3%), white (326, 58.0%) and mid-20s in age (340, 60.5%). Almost half of respondents (47%) reported no prior medical ethics exposure or training in their previous educational experience, while 60% of students across all degree programs reported an interest in more medical ethics education and 92% noted that an understanding of medical ethics was important to their future career. Over a quarter of students (28%) were interested in pursuing graduate-level training in medical ethics, with case-based discussions, small group peer settings and ethics guest lectures being the most desired teaching methods.

**Conclusions:**

The future physician, nursing and physical therapist workforce in our medical community demonstrated an unmet need and strong interest for more formal medical ethics education within their current coursework. Grassroots student-driven curricular development and leadership in medical ethics can positively impact medical education. Subsequent integration of interprofessional training in medical ethics may serve as a vital curricular approach to improving the training of ethically competent healthcare professionals and overcoming the current hierarchical clinical silos.

## Background

With the unabated development of more sophisticated medical technology and the complicated nature of the healthcare system, there is wide consensus that medical ethics education is necessary to develop morally competent healthcare professionals with solid ethical reasoning skills. There is a lack of consensus as to the best pedagogical approach to teach medical ethics, including the content and delivery format of presented material and the teaching method in which students best learn medical ethics [1]. In the past decade there has been a number of reports describing increased student involvement in curricular development, including the development of grassroots medical ethics education with small group peer and near-peer learning strategies [2–4]. At our institution, there was a perceived need for more structured medical ethics education amongst medical students. As a result, a peer-driven grassroots medical ethics curriculum was spearheaded and implemented for interested students who applied for and joined the Leadership Through Ethics (LTE) program [5]. LTE’s focus is a student-driven, small-group based format with faculty mentors exploring important ethical issues and clinical case discussions in medical ethics. This program has been well received by participants to reflect on shared experiences in ethical settings and to gain a more robust, hands-on ethics training in the clinical setting.

Student leaders of this group considered the value of expanding a similar medical ethics curriculum to other disciplines. Through exposure to real-time clinical ethical dilemmas, the LTE students noted a need for a more collaborative, interdisciplinary approach to addressing challenging, morally distressing cases. The students posited that if nurses and physicians learned medical ethics together, difficult ethical cases may be handled better. However, although interprofessional education (IPE) has become recognized as an important teaching method for education curricula in the United States [6], there have been decades of debate about the joint education of nursing and medical students [7–10]. While the current culture of medicine is shifting towards a patient-centered multidisciplinary healthcare system and ethical dilemmas are becoming increasingly complex with modern technology and treatment regimens, medical ethics education curricula have been slow to adopt an interprofessional collaborative environment [11]. By teaching medical ethics to students of different health professional programs in an interdisciplinary and integrative setting, educators can inspire an atmosphere of cooperation and willingness to improve collaboration and communication, mutual respect and shared planning, and decision making in clinical practice [12]. Interprofessional collaboration is an ideal method to explore ethical dilemmas because it allows inclusion for all relevant professional perspectives and encourages collaboration and respect, while also highlighting the strengths of each individual profession’s perspective towards patient care [13–15].

Through implementation of a student-based survey across medical, nursing and allied health professional disciplines, the purpose of this quantitative study was to examine student need and gauge the perceived value for a more integrated medical ethics education curriculum. Additionally, we examined preferences for the format design and curricular components of a graduate level education curriculum. In order to better serve the educational needs and promote interprofessional collaboration among the next generation of healthcare providers, educators must first better understand the differing perspectives among learners in various health professions degree programs in order to guide curricular development. Our findings aim to provide a model for curricular reform that educators and administrators can integrate into the medical ethics education curriculum at our and other institutions.

## Methods

### Study sample

The Medical College of Georgia at Augusta University is Georgia’s state-sponsored center for medical education that includes the School of Medicine (SOM), the College of Nursing (CON) and the Doctor of Physical Therapy (DPT) program in the College of Allied Health Sciences as well as a combined BS/(D) MD undergraduate degree program. We extended an invitation to complete our survey to 1357 students enrolled in the following four programs: 822 students at the SOM (379 first and second year medical students in their pre-clinical education years and 443 third and fourth year medical students in their clinical education years), 370 graduate level students in the CON, 88 undergraduate students in the BS/(D) MD Professional Scholars Program and 77 graduate level students in the DPT degree program. CON students included doctor of nurse practitioner (DNP) students, PhD candidates, and nursing students pursuing a master’s level specialized nursing degree. BS/(D) MD students were undergraduates enrolled in a 7-year program where they will attain a Bachelor’s of Science degree in cell and molecular biology in 3 years and then matriculate to the medical or dental school to obtain a MD or DMD degree. We conducted the study from the beginning of January 2018 to the end of February 2018. The minimum age of participants include in the study was 18 years old. A total of 562 students responded to the questionnaire (41% response rate). Response rates varied from 21% of CON students to 94% of DPT students.

### Survey instrument

The questionnaire included a written informed consent cover letter explaining the nature of the study, voluntary participation and anonymity of respondents. We offered no form of compensation to participants. We used the One45 software system to administer the survey for SOM, Qualtrics to administer the survey to CON and the BS/(D) MD Professional Scholars Program, and an identical paper-based questionnaire to survey graduate level students in the DPT degree program. We chose the method of survey delivery based on the surveying system most familiar to each respective program and included an identical questionnaire with the same written informed consent to all students surveyed. The survey instrument explored (1) respondent demographics and prior education, (2) perceptions about the current level of medical ethics exposure in existing degree programs, (3) attitudes and interest for more formalized medical ethics curriculum, and (4) preferred format design and delivery components among students interested in a graduate certificate or master’s degree in medical ethics. Because of the variable language used to describe medical ethics education in the various health professions, we consistently queried about their exposure to and interest in “medical ethics and/or bioethics education.” For the sake of brevity and consistency we use the phrase medical ethics throughout this text. We gauged student interest in various aspect of medical ethics education using a five-point Likert-type scale ranging from 1‘very unlikely’ or ‘not important at all’ to 5 ‘very likely’ or ‘very important’. The final four survey questions on curriculum format design inquired about respondents’ motivation for pursuing further medical ethics training, preferred curricular design and delivery format, and educational components of interest. These questions were only available to respondents who selected that they were somewhat likely or very likely to pursue graduate level medical ethics training in the preceding question to prevent potentially skewing results with feedback from students not interested in further training. The need for ethics approval was waived by the Institutional Review Board at Augusta University due to its educational purpose, minimal risk status and anonymity of collected data.

### Data analysis

We calculated descriptive statistics including the means, percentages and standard deviations (SD). The t-test and one-way ANOVA with the Scheffe post-hoc test were used to analyze respondents’ characteristics and to examine whether there were any significant differences based on respective educational program. Respondents in the SOM cohort were further divided into students in pre-clinical years and clinical years to examine differences among attitude measures with respect to progression of practical clinical experience and exposure. We conducted all analyses using IBM SPSS Statistics for Windows, Version 25.0 (IBM Corp, Armonk, New York). Graphics are displayed using Microsoft Excel 2019.

## Results

### Demographic characteristics

Demographic characteristics, including anticipated length to graduation, age, gender, race and ethnicity, undergraduate major and previous ethics education of respondents are displayed in Table 1 based on respective educational program. The majority of respondents were female (333, 59.3%), white (326, 58.0%), mid-20s in age (340, 60.5%) and with 2–3 years (297, 52.8%) remaining in their respective degree program prior to graduation. A notable majority of nursing students (32, 40.5%) were over 33 years of age, while all BS/(D) MD students (70, 100%) were under 22 years of age. A large majority of the BS/(D) MD students (63, 90.0%) indicated an Asian race.

**Table 1:** Demographic characteristics of respondents across all educational programs (*n* = 562)

	CON^a^ (*n* = 79)	SOM^b^ (*n* = 340)	BS/(D)MD^c^ (*n* = 70)	DPT^d^ (*n* = 73)	Overall (*n* = 562)
**Anticipated time to graduation, years**					
≤ 1 year	48 (60.8%)	107 (31.5%)	19 (27.1%)	30 (41.1%)	204 (36.3%)
2–3 years	29 (36.7%)	187 (55.0%)	38 (54.3%)	43 (58.9%)	297 (52.8%)
≥ 4 years	2 (2.5%)	46 (13.5%)	13 (18.6%)	N/A	61 (10.9%)
**Age group, years**					
18–22	4 (5.1%)	42 (12.4%)	70 (100.0%)	7 (9.6%)	123 (21.9%)
23–27	24 (30.4%)	255 (75.0%)	0 (0.0%)	61 (83.6%)	340 (60.5%)
28–32	19 (24.1%)	40 (11.8%)	0 (0.0%)	3 (4.1%)	62 (11.0%)
≥ 33	32 (40.5%)	3 (0.9%)	0 (0.0%)	2 (2.7%)	37 (6.6%)
**Gender**					
Female	67 (84.8%)	176 (51.8%)	44 (62.9%)	46 (63.0%)	333 (59.3%)
Male	12 (15.2%)	160 (47.1%)	25 (35.7%)	27 (37.0%)	224 (39.9%)
Gender variant/Non-conforming	0 (0.0%)	3 (0.9%)	0 (0.0%)	0 (0.0%)	3 (0.5%)
Prefer not to answer	0 (0.0%)	1 (0.3%)	1 (1.4%)	0 (0.0%)	2 (0.4%)
**Race/Ethnicity**					
Asian	3 (3.8%)	66 (19.4%)	63 (90.0%)	9 (12.3%)	141 (25.1%)
Black/African American	17 (21.5%)	27 (7.9%)	1 (1.4%)	1 (1.4%)	46 (8.2%)
Hispanic/Latino	1 (1.3%)	13 (3.8%)	0 (0.0%)	3 (4.1%)	17 (3.0%)
White/Caucasian^e^	54 (68.4%)	209 (61.5%)	6 (8.6%)	57 (78.1%)	326 (58.0%)
Other or Mixed Race	4 (5.1%)	25 (7.4%)	0 (0.0%)	3 (4.1%)	32 (5.7%)
**Undergraduate major**					
Bachelor of Science in Nursing	31 (39.2%)	0 (0.0%)	0 (0.0%)	0 (0.0%)	31 (5.5%)
Biological Sciences	13 (16.5%)	241 (70.9%)	63 (90.0%)	12 (16.4%)	329 (58.5%)
Engineering	0 (0.0%)	21 (6.2%)	1 (1.4%)	1 (1.4%)	23 (4.1%)
Liberal Arts and Humanities	10 (12.7%)	27 (7.9%)	0 (0.0%)	1 (1.4%)	38 (6.8%)
Physical Sciences	0 (0.0%)	18 (5.3%)	0 (0.0%)	0 (0.0%)	18 (3.2%)
Other	25 (31.6%)	33 (9.7%)	6 (8.6%)	59 (80.8%)	123 (21.9%)
**Previous ethics education**					
Certification, seminar or workshop training	5 (6.3%)	28 (8.2%)	6 (8.6%)	2 (2.7%)	41 (7.3%)
Former job training	5 (6.3%)	12 (3.5%)	2 (2.9%)	2 (2.7%)	21 (3.7%)
Graduate level course	30 (38.0%)	56 (16.5%)	0 (0.0%)	19 (26.0%)	105 (18.7%)
Undergraduate level course	17 (21.5%)	76 (22.4%)	16 (22.9%)	19 (26.0%)	128 (22.8%)
None/not applicable	22 (27.8%)	168 (49.4%)	46 (65.7%)	31 (42.5%)	267 (47.5%)

^a^College of Nursing, ^b^School of Medicine, ^c^BS/(D) MD Professional Scholars Program, ^d^Doctor of Physical Therapy, ^e^Non-hispanic ethnicity

The majority of students indicated an undergraduate major in biological sciences (329, 58.5%). Students across educational programs reported a varying amount of previous medical ethics coursework or training, with almost half of the respondents (267, 47.5%) reporting no prior medical ethics education or training. A large percentage of SOM (168, 49.4%), BS/(D) MD (46, 65.7%) and DPT (31, 42.5%) students reported no previous medical ethics education, while a majority of nursing students (57, 72.2%) indicated they had some form of previous medical ethics training or coursework.

### Perceived need for medical ethics education

Most respondents (340, 60.6%) across all degree programs endorsed interest in a more formalized medical ethics education curriculum in addition to the medical ethics currently integrated into their respective degree program coursework. Expressed interest was highest among BS/(D) MD undergraduate students (59, 84.3%) and lowest among DPT students (38, 52.8%).

**Fig. 1:**
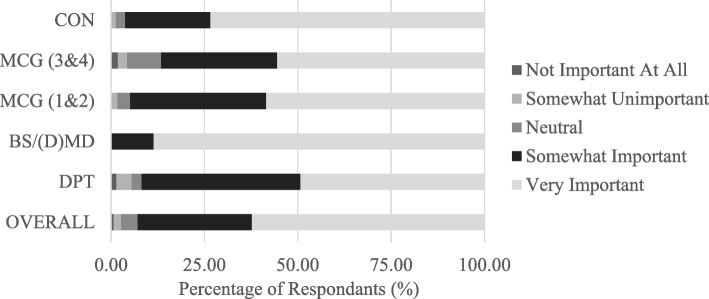
Importance of medical ethics to participants’ future career by educational program (*n* = 562). College of Nursing, CON; School of Medicine, clinical years, SOM (3&4); School of Medicine pre-clinical years, SOM (1&2); BS/MD and BS/DMD Professional Scholars Program, BS/(D)MD; Doctor of Physical Therapy, DPT

Respondents were asked to rate the importance of understanding fundamental principles of medical ethics to their future careers from 1 “not important at all” to 5 “very important”. The majority of students (522, 92.9%) across all degree programs reported an understanding of medical ethics as somewhat (172, 30.6%) or very important (350, 62.3%) to their future career as displayed in Fig. 1. The BS/(D) MD undergraduate students [4.89 (0.32)] placed more importance on understanding of medical ethics to their future career when compared to all other groups (*p* < .001), whereas the DPT graduate students [4.34 (0.84)] displayed the lowest level of importance of medical ethics understanding to their future career (*p* < .001). While there were no statistically significant differences between medical students in pre-clinical versus clinical years, the perceived importance of medical ethics to future physicians declined across progressive cohorts [SOM students in pre-clinical years, 4.52 (0.89) and SOM students in clinical years, 4.36 (0.89)].

While assessing respondent interest in a graduate-level medical ethics curriculum from 1 “very unlikely” to 5 “very likely”, 161 (28.6%) respondents showed interest as being somewhat likely (117, 20.8%) or very likely (44, 7.8%) to pursue graduate level training. As outlined in Table 2, interest in graduate level training was highest among BS/(D) MD undergraduate students [3.19 (1.04)] and lowest among medical students in clinical years [2.49 (1.25)] and DPT graduate students [2.21 (1.11)]. A significant portion of BS/(D) MD undergraduate students (29, 41.4%, *p* < .001) reported that they were somewhat likely or very likely to pursue graduate level medical ethics education, compared to 10 (13.7%) DPT students and 37 (22.6%) medical students in their clinical years.

**Table 2:** Respondent interest in pursuing graduate level medical ethics training by educational program (*n* = 562)

	CON^a^ (*n* = 79)	SOM (3&4)^b^ (*n* = 164)	SOM (1&2)^c^ (*n* = 176)	BS/(D)MD^d^ (*n* = 70)	DPT^e^ (*n* = 73)	Overall (*n* = 562)
**Mean (SD**^**f**^**)**	2.59 (1.30)	2.49 (1.25)	2.82 (1.34)	3.19 (1.04)	2.21 (1.11)	2.66 (1.27)
**Somewhat + very likely**	23 (29.1%)	37 (22.6%)	62 (35.2%)	29 (41.4%)	10 (13.7%)	161 (28.6%)

^a^College of Nursing, ^b^School of Medicine clinical years, ^c^School of Medicine pre-clinical years, ^d^BS/(D) MD Professional Scholars Program, ^e^Doctor of Physical Therapy, ^f^Standard Deviation

### Preferences for medical ethics curriculum

**Fig. 2:**
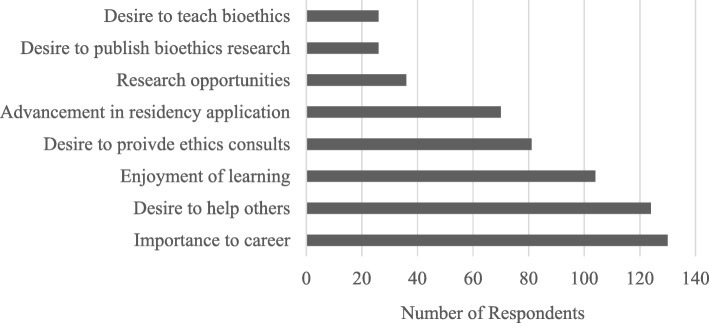
Motivational factors for interest in graduate level medical ethics training across all educational programs (*n* = 161)

Of the respondents (*n* = 161) who reported that they were somewhat likely or very likely to pursue graduate level medical ethics training, the top motivational factors for interest are illustrated in Fig. 2. Importance to career (130, 80.7%) and desire to help others (124, 77.0%) were among the top two motivational factors reported across all programs. Enjoyment of learning (104, 64.6%) and desire to provide healthcare ethics consultation (81, 50.3%) were additional motivational factors for over half of all respondents.

In regard to preferred curricular format, respondents across all programs interested in pursuing graduate level training favored interest in the graduate certification program. A majority of respondents (141, 90.3%) were at least somewhat interested in a graduate certificate program (*n* = 156), and 49 (31.0%) respondents were at least somewhat likely to pursue a master’s degree program (*n* = 158, *p* < .01). CON students preferred a fully online program, whereas SOM, BS/(D) MD and DPT students preferred a hybrid learning format.

**Fig. 3:**
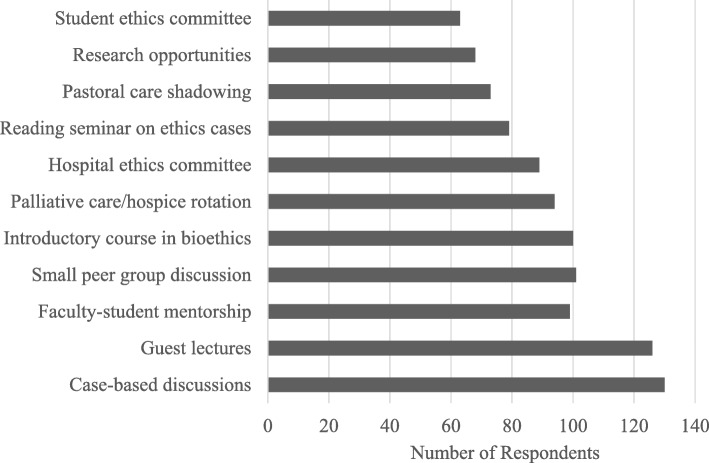
Educational components desired in graduate level medical ethics training across all educational programs (*n*=161)

The top educational components desired among respondents interested in pursuing graduate level training in a medical ethics curriculum are illustrated in Fig. 3. Ethics case-based discussions (130, 80.7%) and ethics guest lectures (126, 78.3%) were among the top two components desired in the curriculum across all respective degree programs. Ethics discussions in small peer groups (101, 62.7%), an introductory course in foundations of bioethics (100, 62.1%), faculty-student mentorship sessions (99, 61.5%), palliative care/hospice clinical rotation (94, 58.4%) and healthcare ethics consultation review committee exposure (89, 55.3%) were additional educational components desired by over half of the respondents.

## Discussion

We report the first survey noted in the literature assessing an interprofessional cohort of students’ perceived need for and interest in further medical ethics education. This survey was inspired by grassroots, student developed medical ethics curriculum [5]. Medical students seeing the value in their experience, envisioned the possibility of such a curriculum being incorporated with other degree tracts, ultimately in an interprofessional format. Our data reveal that nearly one-half of students in health professional degree programs have not received any formal training in medical ethics and over 60% of students from four major disciplines reported a need for further medical ethics education. Over 90% of students across disciplines believed an understanding of medical ethics is important to their careers, and notably nearly 30% of students showed interest in graduate level training. Approximately 90% of respondents interested in further medical ethics training indicated that they were at least somewhat likely to pursue a graduate certification versus 39% indicating that they were at least somewhat likely to pursue a master’s degree. Overall, the results from this study show that the future physician, dentist, nursing professional and physical therapist workforce demonstrate a perceived unmet need for more formal medical ethics education.

There were some notable variations in prior exposure and future interest in medical ethics training, which likely relates to the amount of practical clinical experience of each of the cohorts. Only 28% of CON students had no prior coursework in medical ethics, with nearly 60% taking a course in their undergraduate or graduate studies. On the other end of experience, 66% of undergraduate students had no prior coursework in medical ethics. Medical and DPT students were similar with 49 and 43%, respectively, having taken no prior medical ethics courses. Despite their prior experience, 57% of CON students were still interested in a more formalized medical ethics program. The majority of medical students desired a more formalized curriculum, albeit less of those in their clinical years, 53%, compared to those in their pre-clinical years, 63%. Direct clinical experience likely alleviates the perceived need for more structured medical ethics education. The vast majority of pre-medical/dental students, 84%, with less patient-contact experience in a clinical setting, desired a formalized medical ethics curriculum.

While students reported a broad range of methods desired to teach the educational material, students generally agree on case-based narratives, small group peer discussion and guest lectures as effective teaching methods for medical ethics. Many recent studies support the integration of interactive peer-led, narrative scenarios and case-based small group formats as increasingly popular methods among student preferences to foster experiential learning and problem-solving skills [16–20]. This format allows students to develop practical approaches to solving common ethical dilemmas faced in the clinical setting through open discussion with peers. Furthermore, an educational setting that takes a narrative approach and encourages dialogue specifically in an interprofessional setting has been shown to build upon the three conceptual vertical layers of professional identity, provider-patient communication and interprofessional teamwork [21]. The structure of the LTE curriculum follows this interactive, peer-led, case-based style coupled with experiential learning in medical ethics shadowing, consults and committee participation. It was this adult style of learning that inspired the desire to assess for the need in other disciplines.

An important aspect to consider in the interpretation of this study is not only the diversity in the sample population when making comparisons between the four different health professional programs surveyed, but also the diversity in degree tracts and delivery format within each degree program. For example, CON respondents encompass students in a variety of nursing concentrations including a distance accessible hybrid research-focused doctorate (PhD), a fully online practice doctorate (DNP) and a traditional campus-based clinical nurse leader (MSN) program. In contrast, the BS/(D) MD student respondents are all pursuing a degree in cell molecular biology with a planned matriculation into medical or dental school following their third year of undergraduate studies. By the very nature and teaching format of these individual programs, respondents are exposed to different curricular components and delivery formats which undoubtedly have an influence on students’ perception and attitudes toward medical ethics. Logistics in the development of a more formalized curriculum must be strategically considered to account for the potential difficulty in time constraints and scheduling practicality among students across interprofessional education programs.

Medical ethics education in the health professional curriculum has become a priority among educators and is continuing to evolve. However, the limited time allotted in traditional education curricula leave the content and delivery format of medical ethics education across disciplines open for debate [18–20]. As educators, we are challenged by the significant variation and lack of succinct goals and outcomes in medical ethics teaching methods across institutions nationwide [11, 22, 23]. Studies report effective teaching methods to include further integration of ethics throughout the entire four-year medical school curriculum, small group peer-facilitated discussion, greater than 20 h of undergraduate medical ethics education, and support for an elective ethics course as effective means to increase moral reasoning skills [20, 24, 25].

Our analysis is not without limitations including the relatively low response rate of 41%. This compares with most online surveys and was likely the result of administering surveys in an online format to an already heavily surveyed and busy student group. This was particularly evident among nursing students, who largely were engaged in clinical rotations on satellite campuses away from our home institution. Results may not be generalized nationally as our participants do not represent students from all institutions across the state or country. However, as The Medical College of Georgia at Augusta University is the only state-sponsored medical institution in Georgia, our sample population is likely comparative to a major portion of the future healthcare workforce for the state. Future surveys need to incorporate more direct questions about students’ attitudes specifically towards interprofessional coursework and assessment of IPE impact on learners using educational outcomes and observable behaviors. Despite its limitations, we are encouraged by the survey results which suggest an overall high student-driven interest in medical ethics training.

A year after distribution of this survey, we have developed a 10-credit hour graduate level certification in medical ethics with an emphasis on interprofessional education, which has been initiated for interested incoming graduate health professional students supported by the significant student interest expressed in this study. In its first year of implementation, there are graduate nursing and medical students participating. Curricular components of this certification program include a foundations course for classroom discussion on landmark bioethics literature topics, small group clinical case-based discussions, clinician bioethicists-student mentorship, hospital ethics committee and consult shadowing experiences, mock ethics committee debates, bioethics guest lectures and a capstone bioethics project. Additionally, in order to foster a more interprofessional dialogue on real-time clinical ethical dilemmas, our Center for Bioethics and Health Policy has spearheaded adult and pediatric Bioethics Performance Improvement Teams (BIOPIT) where nurses, physicians, case managers, social workers and other health professionals have a forum to discuss complex healthcare decisions. Other institutions who have developed similar processes have documented decreased moral distress amongst participants [26], and we are currently in the process of studying the effects of the BIOPIT on provider and nursing moral distress. These emerging programs and similar interprofessional efforts will be incorporated into future medical ethics education. Future research will focus on the value of an interprofessional medical ethics curriculum for the different disciplines.

## Conclusions

We report the first study to examine attitudes about medical ethics education among a broad range of learners including pre-medical, pre-dental, medical, graduate nursing and physical therapy students. Our findings from a large interprofessional cohort survey suggests a current unmet need and enthusiasm for expanded medical ethics training. The results demonstrated interest among health professional students across medical, nursing and allied health disciplines for more structured teaching in medical ethics and a desire for graduate level coursework. In order to successfully integrate medical ethics education in an interprofessional setting, it is important to assess the differences in attitudes and preferences among participating students across varying degree programs. Our findings have the potential to guide educational policy and curricular reform regarding training in medical ethics.

As leaders of patient-centered multidisciplinary care, clinician-educators have the ability and responsibility to advocate for enhanced medical ethics education in an interprofessional setting. Our experience demonstrates that student-driven curricular development and grassroots, peer-driven medical ethics education can both inform clinician educators of needs within their respective disciplines and also drive the development of innovative interprofessional initiatives. Future work will aim to evaluate the impact of interprofessional medical ethics education on developing ethically competent future healthcare professionals using high-level educational outcomes.

## Supplementary information

**Additional file 1.**

## Data Availability

The dataset supporting the conclusions of this study is available from the corresponding author upon request at mdefoor@augusta.edu.

## Abbreviations

BIOPIT: Bioethics Performance Improvement Team
BS/(D)MD: Bachelor of Science/Doctor of (Dental) Medicine
CON: College of Nursing
DNP: Doctor of Nursing Practice
DPT: Doctor of Physical Therapy
IPE: Interprofessional Education
LTE: Leadership Through Ethics
MSN: Master of Science in Nursing
PhD: Doctor of Philosophy
SOM: School of Medicine
SD: Standard Deviation
