# Supplementary material for: An interprofessional cohort analysis of student interest in medical ethics education: a survey-based quantitative study
Source: BMC Med Ethics. 2020 Apr 8;21:26. doi: 10.1186/s12910-020-00468-4 (PMC7140336; doi:10.1186/s12910-020-00468-4)
Supplement: Supplementary file 1 — Additional file 1. [file 12910_2020_468_MOESM1_ESM.pdf]

AUGUSTA UNIVERSITY
Institute of Public and Preventive Health

# Assessment of Interdisciplinary Student Interest in a Graduate Level Medical Ethics and Bioethics Curriculum

September 17, 2018

*Prepared for:* Mikalyn DeFoor and Dr. Richard Sams
*Prepared by*:

Data Coordinating Center
Institute of Public and Preventive Health
Yunmi Chung, Ben Ansa, Aaron Johnson

## I. Summary Information

- A total of **562 participants** were included in the analysis.
- Surveys were administered via Qualtrics (nursing and BS/(D)MD students), One45 (medical students), and paper (PT students).
  - 5 participants who completed the survey through One45 indicated they were nursing (n=2) or BS/(D)MD (n=3) students. → Recoded as MCG students
  - 1 participant who completed the survey through Qualtrics indicated he/she was MCG student. → Recoded as BS/(D)MD.
- Data were analyzed using **IBM SPSS Statistics 25**. Descriptive statistics, t-test and one-way ANOVA with Scheffe post-hoc test were used to analyze respondents' characteristics and to examine whether there were any significant differences based on program (CON/MCG/BS-(D)MD).
- Additional analyses were conducted to examine difference between 1st and 2nd MCG students vs. 3rd and 4th.

***Participant Characteristics***

- Participant demographic characteristics by program are shown in Table 1.
- The majority of MCG (49.4%), BS/(D)MD (65.7%), and PT (42.5%) students had no previous bioethics/medical ethics education, while the majority of nursing students had previously taken graduate (38.0%) or undergraduate (21.5%) level courses (Table 1).
- Of the 78 MCG students who reported being a current LTE member, 54.5% (n=42) indicated that the program met their need for more formalized medical ethics education (Table 3). Specific comments are shown in Table 3b. (Many students who are not part of LTE program appeared to have responded to this question).

***Participant Interest in formalized ethics education***

- Most participants indicated that they would like more formalized medical ethics education within their respective programs (61.8%), with the highest level of interest expressed among BS/(D)MD students (84.3%) and the lowest among PT students (52.8%) (Table 2a). Specific comments are shown in Table 2b.

- Students were asked to rate the importance of an understanding of medical and bioethics principles to their future career from "1-not important at all" to "5-very important." The majority of participants (92.9%) reported that an understanding of medical ethics and bioethics principles was somewhat (30.6%) or very important (62.3%) to their future career.

- Interest in pursuing a medical ethics/bioethics graduate certificate or master's program was the highest among BS/(D)MD students (Mean Score: 3.19 ± 1.04). 41.5% of BS/(D)MD students reporting they are somewhat or very likely to pursue a graduate certificate or mater's program in bioethics/medical ethics, compared to 29.1% for nursing and medical students (Table 5). PT students had the lowest level of interest.
    - Of the students who reported they were somewhat or very likely to pursue a graduate certificate or mater's program the top 3 motivational factors for… (Table 6)
        - MCG and BS/(D)MD students were
            1. Importance to career
            2. Desire to help others
            3. Enjoyment of learning
        - CON students were
            1. Importance to career
            2. Desire to help others
            3. Desire to provide healthcare ethics consultation (enjoyment of learning was a close 4$^{th}$, with a difference in n=1)
        - PT students were
            1. Desire to help others
            2. Importance to career
            3. Desire to provide healthcare ethics consultation
    - More students were interested in the Graduate certificate than a master's degree. Nursing students preferred a fully online program, whereas medical, BS/(D)MD, and PT students preferred a hybrid format (Table 7).
    - The top 3 educational components of an ethics curriculum for … (Table 8)
        - Nursing students were…
            1. Ethics guest lectures
            2. Ethics case-based discussions
            3. Ethics discussion in small peer groups
        - Medical students were…
            1. Ethics case-based discussions
            2. Ethics guest lectures
            3. Faculty-student mentorship sessions
        - BS/(D)MD students were…
            1. Ethics case-based discussions
            2. Ethics guest lectures
            3. Introductory course in foundations of bioethics
        - PT students were…
            1. Ethics guest lectures
            2. Ethics case-based discussions
            3. Introductory course in foundations of bioethics

## Contents

I. Summary Information ........ 1
II. Results ........ 4
Table 1. Participant Characteristics by Program, N (%) ........ 4
Table 2a. Interest in a more formalized medical ethics education within respective program, N (%) ........ 5
Table 2b. Comments on having a more formalized medical ethics education within respective program ........ 5
Table 3a. Satisfaction with LTE program, N (%) ........ 18
Table 3b. Comments on satisfaction with the current LTE program ........ 18
Table 4a. Importance of ethics to participants’ future career, N (%) / Mean ± SD ........ 22
Table 4b. Please explain your response, if you indicated neutral-not important at all for Q10 ........ 22
Table 5. Interest in pursuing a medical ethics/bioethics graduate certificate or master’s program, N (%) / Mean ± SD ........ 24
Table 6. Motivational factors for interest in ethics certificate/program, N(%) ........ 25
Table 7a. Program of interest, N (%) / Mean ± SD ........ 27
Table 7b. Program format design of interest, N (%) / Mean ± SD ........ 28
Table 8. Educational components, N(%) ........ 30

## II. Results

**Table 1. Participant Characteristics by Program, N (%)**

| | Program | | | | |
|---|---|---|---|---|---|
| **Characteristics** | **CON** (n = 79) | **MCG** (n = 340) | **BS/(D)MD** (n = 70) | **PT** (n = 73) | **Overall** (N = 562) |
| **Anticipated Graduation Date** | | | | | |
| ≤ 1 year | 48 (60.8%) | 107 (31.5%) | 19 (27.1%) | 30 (41.1%) | 204 (36.3%) |
| 2-3 years | 29 (36.7%) | 187 (55.0%) | 38 (54.3%) | 43 (58.9%) | 297 (52.8%) |
| ≥ 4 years | 2 (2.5%) | 46 (13.5%) | 13 (18.6%) | n/a | 61 (10.9%) |
| **Age Group (yrs)** | | | | | |
| ≤ 17 | 0 (0.0%) | 1 (0.3%) | 0 (0.0%) | 0 (0.0%) | 1 (0.2%) |
| 18-22 | 4 (5.1%) | 41 (12.1%) | 70 (100.0%) | 7 (9.6%) | 122 (21.7%) |
| 23-27 | 24 (30.4%) | 255 (75.0%) | 0 (0.0%) | 61 (83.6%) | 340 (60.5%) |
| 28-32 | 19 (24.1%) | 40 (11.8%) | 0 (0.0%) | 3 (4.1%) | 62 (11.0%) |
| ≥ 33 | 32 (40.5%) | 3 (0.9%) | 0 (0.0%) | 2 (2.7%) | 37 (6.6%) |
| **Gender** | | | | | |
| Female | 67 (84.8%) | 176 (51.8%) | 44 (62.9%) | 46 (63.0%) | 333 (59.3%) |
| Male | 12 (15.2%) | 160 (47.1%) | 25 (35.7%) | 27 (37.0%) | 224 (39.9%) |
| Gender variant/Non-conforming | 0 (0.0%) | 3 (0.9%) | 0 (0.0%) | 0 (0.0%) | 3 (0.5%) |
| Prefer not to answer | 0 (0.0%) | 1 (0.3%) | 1 (1.4%) | 0 (0.0%) | 2 (0.4%) |
| **Race/Ethnicity** | | | | | |
| White | 54 (68.4%) | 209 (61.5%) | 6 (8.6%) | 57 (78.1%) | 326 (58.0%) |
| Asian | 3 (3.8%) | 66 (19.4%) | 63 (90.0%) | 9 (12.3%) | 141 (25.1%) |
| Black/African American | 17 (21.5%) | 27 (7.9%) | 1 (1.4%) | 1 (1.4%) | 46 (8.2%) |
| Hispanic/Latino | 1 (1.3%) | 13 (3.8%) | 0 (0.0%) | 3 (4.1%) | 17 (3.0%) |
| Other or Mixed Race | 4 (5.1%) | 25 (7.4%) | 0 (0.0%) | 3 (4.1%) | 32 (5.7%) |
| **Undergraduate Major** | | | | | |
| Biological Sciences | 13 (16.5%) | 241 (70.9%) | 63 (90.0%) | 12 (16.4%) | 329 (58.5%) |
| Other | 25 (31.6%) | 33 (9.7%) | 6 (8.6%) | 59 (80.8%) | 123 (21.9%) |
| Liberal Arts and Humanities | 10 (12.7%) | 27 (7.9%) | 0 (0.0%) | 1 (1.4%) | 38 (6.8%) |
| Bachelor of Science in Nursing[a] | 31 (39.2%) | 0 (0.0%) | 0 (0.0%) | 0 (0.0%) | 31 (5.5%) |
| Engineering | 0 (0.0%) | 21 (6.2%) | 1 (1.4%) | 1 (1.4%) | 23 (4.1%) |
| Physical Sciences | 0 (0.0%) | 18 (5.3%) | 0 (0.0%) | 0 (0.0%) | 18 (3.2%) |
| **Previous Ethics Education** | | | | | |
| Undergraduate level course | 17 (21.5%) | 76 (22.4%) | 16 (22.9%) | 19 (26.0%) | 128 (22.8%) |
| Graduate level course | 30 (38.0%) | 56 (16.5%) | 0 (0.0%) | 19 (26.0%) | 105 (18.7%) |
| Certification, seminar, or workshop training | 5 (6.3%) | 28 (8.2%) | 6 (8.6%) | 2 (2.7%) | 41 (7.3%) |
| Former job training | 5 (6.3%) | 12 (3.5%) | 2 (2.9%) | 2 (2.7%) | 21 (3.7%) |
| None/not applicable | 22 (27.8%) | 168 (49.4%) | 46 (65.7%) | 31 (42.5%) | 267 (47.5%) |

Abbreviations: CON – College of Nursing, MCG – Medical College of Georgia, BS/(D)MD – Professional Scholars Program

[a] This option was only shown on the Qualtrics survey and not on One45

**Table 2a. Interest in a more formalized medical ethics education within respective program, N (%)**

| | Program | | | | |
|---|---|---|---|---|---|
| | **CON** (n = 79) | **MCG** (n = 340) | **BS/(D)MD** (n = 70) | **PT** (n = 73) | **Overall** (N = 562) |
| **Q8. Would you like more formalized medical ethics education within your respective program?** | | | | | |
| Yes | 45 (57.0%) | 198 (58.2%) | 59 (84.3%) | 38 (52.8%) | 340 (60.6%) |
| No | 34 (43.0%) | 142 (41.8%) | 11 (15.7%) | 34 (47.2%) | 221 (39.4%) |

| | Program | | | | | |
|---|---|---|---|---|---|---|
| | **CON** (n = 79) | **MCG[3,4]** (n = 164) | **MCG[1,2]** (n =176) | **BS/(D)MD** (n = 70) | **PT** (n = 73) | **Overall** (N = 562) |
| **Q8. Would you like more formalized medical ethics education within your respective program?** | | | | | | |
| Yes | 45 (57.0%) | 87 (53.0%) | 111 (63.1%) | 59 (84.3%) | 38 (52.8%) | 340 (60.6%) |
| No | 34 (43.0%) | 77 (47.0%) | 65 (36.9%) | 11 (15.7%) | 34 (47.2%) | 221 (39.4%) |

**$\chi^2$ = 23.097; p< 0.0001**

**Table 2b. Comments on having a more formalized medical ethics education within respective program**

| **Program** | **Q8 = 1 (Yes)** | **Q8 = 2 (No)** |
|---|---|---|
| **CON** | • "As an ICU, I have witnessed situattions such as a patient being kept on life support for an extended period of time (weeks to months) with no end in sight. This coupled with the fact invasive procedure were still being ordered would have called for an ethics committee which the institution lacked at the time.<br>• Medical ressources are not unlimited. People want to die with dignity. Medical ethics is absolutely important."<br>• "As healthcare continues to evolve, there are new issues that will continue to come up. A most recent example is an elderly man with ""do not resusitate"" tattooed on his chest, when he had cardiac arrest and taken to the ED this caused confusion and controversy...would this tattoo serve as an appropriate advanced directive??<br>• Things like this will only continue to come up in the future"<br>• Ethics is spread so thin among the courses that I have taken so far that I think it would be a good idea to have a class that specifically talks about ethics and ethical situations that may arise. As nurse we need to be capable to handle these type of situations and not fall short in our total care.<br>• Having a formal medical ethics education for students that want to participate in research of any kind would be very helpful. I have previously been a research assistant and having that form of education would be helpful in furthering our careers.<br>• I believe that an online program covering medical ethics (3 hours or less) would be beneficial. Although I'm sure there's enough content to have a medical ethics class, I think graduate nursing students with over 1 year of nursing experience should be exempt from the course. I have been trained in medical ethics twice before.<br>• I do not really understand what medical ethics is. | • Ethics in Nursing course was sufficient<br>• Ethics is included in many of the classes I have taken at AU. From previous experience (where ethics was taught as a separate class at another university), I have found that including ethics into the regular curriculum makes it a more applicable topic, as opposed to separating it out into its own distinct course.<br>• I believe the bioethics class I took in this program provided me with a sufficient amount of baseline/general information on the principles of bioethics. |

| Program | Q8 = 1 (Yes) | Q8 = 2 (No) |
|---|---|---|
| | • I feel like this is a huge issue for practicing nurses and we are not taught how to navigate bioethics in a realistic way.<br>• I feel that, as a future nurse, it is very important to fully understand medical ethics in order to properly advocate for our patients. It is also important to be aware of potential ethical issues, to know how to spot them, and to know how to solve certain ethical issue that may arise in medical practice.<br>• I realized last semester when I took a class on health policy and bioethics how important medical ethics are in the 21st century world. From new technologies to changing policies to widespread disparities, more graduating healthcare professionals with a background and understanding of standardized medical ethics can help change a medical society that is stuck in outdated and unproven methods of caring for patients.<br>• I think a medical ethics class is imperative to anyone in the medical field. Thus far, in our program (DNP-FNP), we have not come across any ethics class. I don't see it on our schedule but our cohort still has 1 more year (4 more semesters counting this one) to go and it would be beneficial to have a class on medical ethics even if it is one credit. To ask us to have a post master's certificate in medical ethics (or some such thing) is going to guarantee that we will not get an ethics class due to time constraints, taking boards, etc. - but if it is already part of the curriculum then it would ensure enough exposure to it. Thank you for the chance to speak up through this survey - I will say this again - everyone in the medical field needs a medical ethics class. But please if you are going to do it, put the right professor(s) in place for it to take away from the stress level that is already in place throughout our respective programs. Thank you!<br>• I think it would be a valuable tool in my nursing career to have as much ethics education as possible.<br>• I think this education will benefit all healthcare students.<br>• I think with the advances in the field of genetics as it relates to editing DNA with for example the CRISPR technology and all of the advances that will be made along those lines in the future, a concentration and study on how this and other areas impacts patients now and in the future is a necessity. I would be interested myself in such a curriculum. I feel that exploring the potential impacts this field and others could have on our patients is a part of our duty as healthcare providers.<br>• I would definitely like a medical ethics course especially with emphasis on professionalism in the hospital and on social media (like your social media should not have antiabortion or anti gay stuff that may make a future patient that doesn't really know you personally feel uncomfortable with you as their nurse)<br>• In the medical field, we deal with ethic dilemmas everyday and would like to have more formal training to be prepared for various situations to response appropriately while providing high quality care.<br>• It is an important aspect of patient care.<br>• It is an important topic that we must be aware of today and a formal course could ensure that we are fully informed.<br>• It's a growing concern in Nursing field.<br>• Medical professionals are faced with ethical dilemmas every day. Exploring medical ethics deeper broadens our understanding and ability to examine these events as they occur in our professional lives.<br>• Not sure what it would entail<br>• The design of the class was a team based learning course. Everything was online and very virtual. I would prefer an in class design where relationships are formed in person and communication is direct with out technology .<br>• The ethics education that I received in undergrad was very brief and did not cover many issues that I currently see in my full-time job (emancipated minors, EMTALA, consent for treatment, etc.) | • I feel as though it is embedded in our coursework throughout the program.<br>• I feel the one class that we did have was adequate and enlightening for the DNP program. We discussed different cases including the Henrietta Lacks case and had relevant discussions.<br>• I feel there is enough work. I'm not a researcher and I feel that specific training would have more benefit in that field. Yes, bioethics does apply to nursing but I feel the priority is to gain a foundation of nursing skills, assessment, and practice- not feature a component of bioethics.<br>• No, as I feel that our current curriculum covers ethics in a relatively thorough manner.<br>• Our program offers a great course within the curriculum to address ethics in research.<br>• The current program has enough in the program that addresses this.<br>• To be honest it just adds to a challenging course load<br>• We have a class devoted to medical ethics |

| Program | Q8 = 1 (Yes) | Q8 = 2 (No) |
|---|---|---|
| | • To clarify, I would like to see bioethics and nursing ethics more so than medical ethics. If there is an ethics certificate program, I would very much like to get the certificate. I would also be interested (because I am also a faculty member) in devising curriculum and teaching in an ethics certificate course. I have undergraduate training in feminist ethics and copious relevant graduate training in philosophy, nursing, history, and feminism.<br>• We are doing one module in CITI training (either 2 or 7) this semester and perhaps will be doing more in the following year. It would be beneficial to have a medical ethics course that also teaches end of life care in our curriculum.<br>• Would love to learn more!<br>• Yes, these issues come up a lot in healthcare. Many people do not know how to address these issues. | |
| **BS/(D)MD** | • As an aspiring healthcare provider, it's important to have a knowledge on ethics.<br>• ethics is often taught but never truly understood by many people<br>• Have not been exposed to much about medical ethics education as an undergraduate<br>• I believe that medical ethics is just as important as the scientific aspects of medicine!<br>• I believe that receiving more formalized medical ethics education would raise awareness on this aspect of being in the healthcare field and deepen our understanding and appreciation of how it affects our future prospective careers.<br>• I feel as though medicine is moving to be a more litigious field and a comprehensive understanding of bioethics and medical ethics could help ease the transition.<br>• I feel that the information would be useful in the professional setting.<br>• I strongly suppport instituting a medical ethics program due to the rising need for healthcare providers that are aware of the individual cultural background of each patient and able to incorporate that background into their care.<br>• I think it's an interesting topic that is very applicable for future career goals. Medical ethics is an important topic to consider and be knowledgeable in when working in the healthcare field.<br>• I think it's important that we have some sort of medical ethics education at one point during our education because that is what we will be dealing with on a daily basis once we graduate and we will need to know how to deal with different situations.<br>• I think it's important to understand what may be considered ethical in different situations since ethics is so subjective. If it's possible to fit it into some sort of curriculum, I think it'd really help students with their professions later on.<br>• I think medical ethics is an essential tool for any future dentist or doctor to have. It combines ethical philosophy with core natural science, making it not only a unique subject but also a worthwhile one.<br>• I think that it's important for students that want to pursue careers in the healthcare field be exposed to the ethical issues in the profession so that they know how to act appropriately if faced with those situations.<br>• I want more education on bioethics.<br>• I would like more information and knowledge on what to do in more "grey" area type of problems<br>• I would like to learn more about medical ethics as it directly applies to my future profession.<br>• I would like to take a medical ethics class in undergraduate school.<br>• It is important to know what is ethical and what is unethical so that one can make decisions that are well-thought-out.<br>• It would be an interesting topic to explore and would be helpful in this volatile world. | • I believe we take this course later, so I can't decide until then.<br>• That's more of a graduate level thing because then there is more context |

| Program | Q8 = 1 (Yes) | Q8 = 2 (No) |
|---|---|---|
| | • It would be beneficial for people who want to enter the field of medicine to be familiar and knowledgeable about medical ethical issues<br>• Medical bioethics is important for undergraduate students to understand especially in the light of changing medical views.<br>• Medical ethics education seems like an interesting topic to learn before entering medical school or the medical field.<br>• Medical ethics is a really hard concept to grasp at any stage of education. As a doctor, it is really important to understand the different situations and their different ethical implications.<br>• Medical ethics is an imperative part of the medical curriculum and I would like to be exposed to more issues so I can make better decisions in the future career.<br>• Medical ethics is an important field for those who plan on going into medicine.<br>• Medical ethics is interesting to me, and something that I would like to explore to help inform my own ethical practices down the road. Although I am not sure that I would be able to commit to much into it.<br>• Medical ethics is something that is important for doctors and patients alike. Students need to learn about them in order to be a proper medical professional and to learn about what principles are widely accepted by society and what is not accepted.<br>• Medical ethics is very important in terms of knowing how to balance how to care for a patient while at the same time respecting any beliefs or practices that a patient has. Since I feel that at times this part of medicine gets lost (or is even nonexistent at times) in the curriculum, it would be nice to have more formalized education to prepare us to be not only the best but also the most prepared physicians that we can be.<br>• Medical ethics is very important in the field of medicine. People involved in the medical field are faced with ethical dilemmas on a day to day basis and it is important to develop the skills necessary to make quick and ethically proper decisions early on.<br>• The ethics class we took was not medical in nature at all. We wrote poems and read articles, and while being able to use proper verbal and nonverbal communication is very important in the medical field, the capacity at which we learned in class did not directly apply to medicine, let alone possible medical scenarios.<br>• There is a specific importance in ensuring that the student has had one bioethics course. The ability to know how to deal with the social and ethical problems the surround becoming a doctor are significant to the overall success of the individual.<br>• This is a pressing issue today and is also tested on the mcat, thus highlighting it's importance.<br>• Yes, I think it would be extremely beneficial to learn about bioethics.<br>• Yes. It's important to get exposure to our future goals and professional aspirations | |
| **PT** | • A brief summary and need to know information will be beneficial in the real world setting<br>• because there is a large responsibility for not just the patient's body but their entire being, it should be discussed more.<br>• I am very intrigued by the death and dying process and how the physical therapist can: 1) be utilized optimally 2) personally be comfortable, and 3) be comforting to the patients and related caregivers.<br>• I think a course in medical ethics would help me when I get out in the working world.<br>• I think students are too caught up in academic material that they forget how ethics play a huge role in healthcare. | • Have a good understanding currently through program. Enough for me.<br>• I already have a strenuous program with a heavy wkld I wouldn't have time for additional courses. |

| Program | Q8 = 1 (Yes) | Q8 = 2 (No) |
| --- | --- | --- |
| | • I think that it could be very applicable to the workforce (but also don't want to extend my program length)<br>• I think this is very important for my medical education & I believe it's important for all healthcare professionals to learn about ethics.<br>• I think this would be beneficial if it could be incorporated into the existing curriculum.<br>• I would appreciate integration of medical ethics into the current curriculum.<br>• If we could incorporate ways to handle ethical issues/what they are throughout PT curriculum<br>• it could be useful to know<br>• It could only be beneficial to learn how to better act as a healthcare provider. Learning proper ethics would encourage a more professional and effective environment throughout my career.<br>• It is important<br>• It is important to be educated on medical ethics to be able to treat our pts. ethically to educate them on their rights, and have knowledge to back it up.<br>• It is something very important in our field & would be great to actually have a course or section of a course related to it.<br>• It might be good to have it woven more throughout the curriculum.<br>• It would be great to learn more about serving people of different cultures. Also specifically w/ working with patients in 'tricky' situations (eg. uncomfortable palpation sites or males working with females).<br>• It would be helpful to the making of decisions in my chosen career<br>• It's such a complex area - more instruction would be beneficial<br>• Patient population is ever changing and everyone seems quick and happy to sue healthcare providers. further education would be valuable.<br>• To protect ourself in the workplace<br>• Very important topic, especially in the world today.<br>• We go through generalized medical ethics in the PPE courses. Expansion within these courses may be beneficial.<br>• Yes, perhaps our program can be focused on ethics w/ regards to different cultures and with women. | • I believe that the topic of ethics is touched upon within our curriculum, but cannot imagine a bigger chunk of the class being dedicated to ethics. I believe the day that Reverend Flowers came in to talk to us was very eye opening, though.<br>• I do not desire more fluff than we already get.<br>• I feel like I get enough education on this interspersed amongst a variety of my other courses, such as Professional Practice Expectations.<br>• I feel like my program adequately covers this<br>• I feel like our program adequately covers a basic level of ethics that would adequately guide me in ethically questionable situation<br>• My program does well discussing ethics<br>• My program incorporates ethics into the curriculum very well.<br>• No.<br>• our course load is heavy enough already<br>• Our program does a sufficient job of addressing ethics<br>• The curiculum already touches on these aspects throughout our 3 years here<br>• threaded in our curriculum already<br>• We touch on ethics multiple times in our curriculum<br>• What is it? |

<table>
<tr><td></td><td colspan="3">Comments</td></tr>
<tr><td></td><td colspan="3">Note: MCG student survey was collected using One45 and the comments were not linked with the respective yes/no question. Therefore we were unable to examine the comments by responses to Q8. Instead, we sifted through the comments and categorized into 3 groups: positive, neutral, negative.</td></tr>
<tr><td>MCG</td><td>Positive</td><td>Neutral/Suggestions</td><td>Negative</td></tr>
<tr><td></td><td>• I want more education on bioethics<br><br>YR 1-2<br><br>• I think it would be good to understand the decisions that physicians are often required to make and how they go about making them.<br>• I feel that the few ethics discussions we have throughout the first and second years of medical school are not enough exposure to allow us to feel confident in tackling ethical dilemmas in our careers.<br>• Ethics is an important part of Step 1, and as the exam gets closer and I do more and more questions I realize it's an area we did a poor job of covering within the curriculum.<br>• I think that all physicians should gain exposure to medical ethics during their pre-clinical years. A classroom setting allows students the time for careful reflection and discussion of difficult topics before they have to confront them in practice. The classroom allows students to process ethical theory before they have to make ethical decisions that impact patient care. If students have not been asked to address these questions in advance to clinical rotations, they will feel uncomfortable addressing them in practice and could make more errors in clinical judgment as a result.<br>• While our inter sessions are wonderful and informative, they lack a more formal bioethics curriculum, which I think is key to making ethical medical decisions in the future.<br>• Yes, it is an important facet to my future profession.<br>• I applied for the Leadership Through Ethics program and was not accepted. The first-year medical school class did not even fill the spaces, so I am upset that I was not able to participate</td><td>YR 1-2<br><br>• It's never done well in a way that's not invasive in our schedule, or engaging to large groups. Ethics can only be interesting to me in small groups, where free discussion is possible.<br>• Replace journal club with it.<br>• I believe the ethics lessons we have that are integrated throughout the module are great insights into the ethics side.<br>• I think we have enough, the only thing I would be interested in would be real work examples and resolutions that actual physicians have encountered.<br>• I honestly don't know. Medical ethics seems to me to be about critical thinking and view points and while it is good to engage people to think critically and examine all sides of an issue, how much of that could be "taught" is</td><td>YR 1-2<br><br>• I feel a formal course/class beyond a lecture here or there would not be helpful, and would take away from the limited time we have to prepare for the boards. Important topics to cover with future physicians are our resources to contact if we have a issue regarding ethical treatment for our patients when conflict arises, and basic expectations the medical community/society holds regarding common ethical issues. (I believe many of these topics are already being covered in our case discussions we have each module.)<br>• I feel that a lot of medical ethics is intuitive, and the parts that aren't are delineated by company policy and vary between institutions (with a few exceptions).<br>• I think that a lot of ethics is best determined during practice. Talking about it is nice but during practice i think it'll be different.<br>• I think at MCG we are too busy with our own coursework to fit in more information about ethics. Also, outside of examples that guide us how to act in certain situations, I don't think anyone's ethical beliefs are changed in the discussions.<br>• it is a waste of time, that we could be using more productively.<br>• I think medical ethics is very interesting to talk about and discuss, but in all of the discussions that I have participated in, the same arguments always get rehashed and the same conclusions are always proposed. I think that it's important to mention potential scenarios and to give a basic foundation in order to help us as developing physicians, but a more robust ethics course should not be required.</td></tr>
</table>

| | Comments | | |
|---|---|---|---|
| | because I actually wanted to learn more.<br>• Having discussions over relevant topics to keep us all up to date with where medicine is leading towards.<br>• I think that by working in the medical field we are exposed to a lot of ethical scenarios and we need to make sure we make the right decision<br>• I greatly appreciate the medical ethics cases that are reviewed in the first and second year curriculum at MCG. I feel that by expanding these cases to a monthly 1-2 hour course could be extremely helpful in solidifying the importance of these topics.<br>• It'd be good to have it for those interested in it.<br>• I would like to incorporate more medical ethics courses in our curriculum. I understand that LTE does great work with this, but it would be nice if students who were not involved could participate in the activities that those members could.<br>• Entering the medical field, it seems like an important topic to be well-versed in. And where else would I learn this topic than in medical schoool?<br>• we learn all about normal and abnormal biology but we rarely learn about the ethics of medicine and there has been lots of cases associated with medical ethics<br>• I think the option should exist for all students in the medical field at Augusta University to get more accustomed to medical ethics in a formal way. We are setting ourselves up for failure if we continue on to our careers without a firm and formal understanding of how we fit into the medical landscape. Without this, we are operating off of the guidance from our elders in the medical field - potentially bringing their ethical errors into the future.<br>• It would be helpful to learn how to have more conversations with patients and ethically approach difficult situations.<br>• I think it would be beneficial to make we have all looked at them at least once.<br>• I'm interested in working in academic bioethics, and a formal curriculum would help me do that. | | • doubtful.<br>• It is already integrated into the curriculum pretty well. We already have a lot on our plates, and it would be difficult to add something else into the program.<br>• Medical ethics seems to be intuitive and based on being a good person that respects the wishes of the patience. I don't think a formalized course is needed to teach that.<br>• We have ethics case discussions in our schedule.<br>• Not interested in formal education in bioethics, I feel this is something I can learn on my own |

| | Comments | | |
|---|---|---|---|
| | • I would enjoy more than one intercession on discussing ethics.<br>• I believe a deep understanding of ethics is crucial to practicing medicine or any health care field as well as possible. Especially as technology advances and gives us more power and choices than we've ever had before, it is important to always consider all the ethical implications of our actions. While the occasional lecture or group discussion of ethics within our regular education is better than nothing, these classes are often ignored or skipped because students would rather focus on what is actually on their test. Having a formalized program would give students an actual goal to work for and would justify their involvement in ethics education activities.<br>• I enjoy learning about this topic as it will be applied to our careers in the future on a daily basis.<br>• Medical ethics plays an important role in my medical career. Since hospitals have ethics committees, it is imperative as a future physician to understand basic concepts of medical ethics.<br>• Ethics are extremely important in medical practice, keeping in the practice of making ethical decisions improves our healthcare system as a whole.<br>• I think it would definitely be a beneficial element to incorporate into our medical studies so that we learn to consider ethics almost as an instinct for the future.<br>• It would be helpful to incorporate more ethical scenarios and modes of thought into medical education, as this will undoubtedly appear in our practices<br>• I view it as important for students to understand the ethical background of the field in which they are entering.<br>• Medical ethics are continually evolving so it would be nice to receive instruction on how to navigate such a complex field in our future careers.<br>• I would like more education in medical ethics because ethics are an integral part of medicine that we as medical students receive very little instruction.<br>• I would like to learn more about biomedical ethics and ethical problems that physicians face, similar to | | |

| | Comments | | |
|---|---|---|---|
| | scenarios we were given during Art of Doctoring.<br>• I think it is important and something that I will have to deal with from time to time.<br>• It's so important in medicine! And we have very little training in it. I don't know how we can expect to be competent physicians who are trying to promote the overall health of their patients if we don't have ethical training.<br>• It would be nice if we could have a more extensive Bioethics intersession at MCG. I feel that the one or two days we learned about it was not enough since it will be so critical to our future professions.<br>• Physicians are faced with many ethical dilemmas and the more training we can receive will only lead to us being better prepared in the future.<br>• We had some ethics discussions at the beginning of the year, but I wish we had more periodic discussions in smaller groups about ethical principals. Ethics is very important to the field of medicine and it is important that medical students receive adequate medical training.<br>• Anyone in the healthcare field will come across ethical dilemmas yet the only "exposure" we receive are little sessions maybe once a semester or year during a break between modules (at least for MCG). When we go to clinical years, I think we could be better prepared for those dilemmas rather than be stunned in the moment only for the situation to worsen because we were not adequately prepared.<br>• I would be interested in a certificate program or bioethics Master's degree completed concurrently with the M.D. program. I'd like this to include curriculum components that are primarily practical experience and discussion based augmented with minimal didactic study. Additionally, such a program should offer ethics research opportunities in a formalized way.<br>• I would like a breakdown of current bioethical thought and law, including case-law and precedents. A certificate or dual degree program would be nice. | | |

| | Comments | | |
|---|---|---|---|
| | • I would like more resources to use when I need to look up ethics related articles.<br>• I think medical ethics should be a requirement in our medical curriculum, or at least more formalized, because it is something that we will inevitably experience. It is important to get us to think about certain scenarios where our ethics/morals<br>• I think learning about medical ethics is not only really interesting but important to a career in medicine, as physicians need to make tough decisions all the time in very short periods of time occasionally.<br>• An ethics certificate and program would be great. The medical field is always making rapid advancements, and it's important our medical ethics always keep pace with those advances.<br>• I think ethics is just such an important aspect of every medical decision<br><br>**YR 3-4**<br><br>• More formal opportunities to shadow or participate in medical ethics on campus in the hospital would be nice.<br>• Yes, as a member of the LTE program, I feel that the ethics training incorporated within this program really should be integrated within the MD program itself. Many of my classmates turn to me when they face ethical dilemmas at the hospital, because we don't expect to see them, and then once we're here, they are an everyday part of life.<br>• Medical ethics is not always a simple topic. I would like more formalized education to feel more comfortable when handling complex situations with future patients.<br>• I think offering elective courses in medical ethics or offering a certificate in ethics would be beneficial.<br>• We have several intersessions devoted to medical ethics that are quite well run.<br>• There are many instances that are not discussed in the first two years of medical school that come up during third year or in observing how healthcare team members interact with each other. It would be helpful to have | **YR 3-4**<br><br>• I feel that our program already incorporates a fair amount of medical ethics discussion. Adding more would require detracting from the medical science curriculum which I feel we already struggle to fit in to our allotted time anyway.<br>• I would like to have more ethics discussions, however, NOT at the expense of the scientific curriculum at MCG.<br>• Medical ethics is important but hard to teach in didactic format or through MCQs. Case-based learning and discussions seems effective but is difficult to arrange | **YR 3-4**<br><br>• This is a voluntary study, yet there is no way to opt out to get the study out of our one45 to do's. Please edit the study designs.<br>• We covered a lot on this topic already in medical school.<br>• Ethics is far greater learned in small, informal discussion groups. Unfortunately due to class size, and the need to standardize education amongst groups, this is impractical. Additionally, I think I have had several opportunities to personally experience cases involved medical ethics while on my clinical rotations, and this has been far more beneficial to my learning than an additional formal education program would be.<br>• Ethics is something you either have or you don't.<br>• I feel like you can't "teach" ethics, maybe just let students know how the ethics committee can be contacted.<br>• I feel that our program already incorporates a fair amount of medical ethics discussion. Adding more would require detracting from the medical science curriculum which I feel we |

| | Comments | | |
|---|---|---|---|
| | more formalized group discussions to help us be more prepared to discuss differences in opinions.<br>• yes in a limited capacity<br>• I enjoy medical ethics and believe that it should be a required part of the medical curriculum<br>• Yes, it would have been nice to have smaller workshops regarding medical ethics.<br>• prior to third year of med school your ethics must be staunchly in place before they are tested<br>• I always love to learn what potential ethical situations I may run into during my career. It is my belief that the more you understand ahead of time the easier it will be to know how to respond in the moment with less hesitation.<br>• I think a basic understanding of medical ethics can be useful to all medical professionals. We have some basic training during some of our intersessions, but a more former refresher may be useful.<br>• Providing an ethical foundation as part of medical education would be welcome. I don't think a bad job is being done now.<br>• I think its important to understand that ethics and morality are grounded in objective standards that are not dictated by societal norms. For example, if society decides that babies are a delicacy, do we start eating them because it's trendy? The answer to this is grounded in ethics and morality, and that is why it is important to study.<br>• During our clinical years, we're often left out of the discourse if a situation with a patient becomes ethically complicated. I think we should be taught through the patients that require that thinking.<br>• I think Ethics is an important aspect of any medical career and one that is frequently not taught as much as the other sciences.<br>• Would be beneficial to medical career<br>• Can always get more!<br>• I think that a lot of students have questions regarding the ethics of things we do/see in our medical system, but we don't have a venue for discussing them. Often times, we feel discussion | and resource-intensive given the constrains on medical education already.<br>• I would like to receive more ethics education if more depth is covered, but so far, all medical ethics training I have received has been redundant.<br>• The medical partnership has many lectures designed to present medical ethics to the student. I didn't have any undergraduate courses in this, so I enjoyed getting these lectures in medical school.<br>• Medical ethics is welcome in the curriculum, but I don't necessarily desire more "formalized" instruction in this area.<br>• Medical ethics needs to be more emphasized in medical curriculum. Currently, there is intercessions, but there needs to be yearly and more organized training. I don't believe there is time or need for a certification during medical school.<br>• Depend on the nature of the course. Ethics for some cant be taught. People need to learn how to feel and use their best empathic | already struggle to fit in to our allotted time anyway.<br>• I think MCG already does a good job of incorporating medical ethics into our curriculum. There is also an opportunity to participate in a leadership through ethics program.<br>• I think medical ethics can be learned better in practice than within a formalized educational setting. I've learned a lot more about medical ethics a little over halfway through my 3rd year clinical rotations than I ever did in 1st or 2nd year through formalized education.<br>• I thought the medical ethics curriculum at the AU/UGA Medical Partnership (MD program) was more than adequate.<br>• I feel like the medical school in Athens covers medical ethics well<br>• we recieved biomedical ethics training during our graduate program<br>• I would rather trach and Peg myself<br>• Time need not be wasted in the classroom when the best way to learn about these ethical principles is on the job, in the real world, with real world situations.<br>• We have enough stuff in our curriculum that wastes time and doesn't prepare us for USMLE Examinations<br>• There is only so much time to teach the medical curriculum in a streamlined, high yield format that to dedicate more time to medical ethics, while it may be beneficial, may negatively affect the ability to teach students what they really need to know to score well enough on the USMLE exams to compete with MD, DO, and Caribbean students across the country. This kind of education should be focused on residents, or should be done as online modules.<br>• There is already an established medical ethics discussion during phase 1 and 2 courses.<br>• We learn about ethics in medicine through everyday practice and common sense. In residency, I think it would be useful.<br>• I believe we had a rudimentary but adequate amount of formal teaching regarding medical ethics. In my opinion |

| | Comments | | |
|---|---|---|---|
| | with teachers/attending in the field may looked up on as being defiant, so we don't say anything. Providing a safe-space venue to learn, talk, and think about these topics would have been an incredible outlet during medicial school<br>• We only had a few days of formal ethics education and training. It would be great to be able to talk to medical professionals about common ethical dilemmas that we might encounter during our careers and to learn about how they dealt with them. We only got a lecture similar to this long before we started our clinical training.<br>• I think a formal lecture series on medical ethics, possibly integrated into our "art of doctoring" intercession, would be very beneficial for our medical education. In any field of medicine we pursue, I think it is important for physicians to be comfortable with the ethical dilemmas that will likely occurs during their practice.<br>• It is an important issue that needs to be presented methodically and critically, to train us to think critically about ethical dilemmas.<br>• I think many students have a good moral compass however a formalized curriculum with discussion of more challenging ethical questions is an important part of medical training<br>• Understanding medical ethics is very important as a physician and formalized training would help me to feel more proficient with handing medical ethics. I would also be interested in being on a medical ethics committee but wouldn't feel comfortable with it unless I had formalized training.<br>• It is my observation, from my experiences during the clinical years, that most medical ethical decisions seem to made through emotion, or what "feels right." I would be interested to learn if there are more formal approaches to making ethical decisions in the hospital. I am also believe that communication, specifically patient/doctor or family/doctor communication is at the root of most ethical dilemmas in the hospital, and many conflicts could be resolved by improve communication | judgement. There is no right answer.<br>• Medical school is too busy already. I think formalized medical ethics should be an elective for interested students. | taking more time away from the basic and clinical sciences would not be in the best interest of the students and their future patients. The principles of medical ethics are easily understood by most all of the students due to the fact that we have been brought up to be moral and are entering a career in service to our fellow man. The students who do not have these ethics ingrained on their hearts should not have been accepted to medical school. I think the admissions department, for the most part, is doing a fine job of curating a diverse group of students who are of high moral character. |

| | Comments | | |
|---|---|---|---|
| | strategies. This would be very useful to understand.<br>• Medical ethics is an intrinsic component within medicine and would benefit greatly from more formalized training especially before getting to the wards in 3rd and 4th year.<br>• It is good for medicine<br>• Ethics education related to how to evaluate and respond to ethical challenges in the hospital. | | |

**Table 3a. Satisfaction with LTE program, N (%)**

| | |
|---|---|
| | **Overall** (N = 78) |
| **Q9. If you are currently a member of LTE, do you feel that this program is meeting your needs for more formalized medical ethics education?** | |
| Yes | 43 (55.1%) |
| No | 35 (44.9%) |

*Note: Many students who are not part of LTE program appeared to have responded to this question. There are a total of 54 members in LTE (all MCG students), however 78 MCG students responded to this question.*

**Table 3b. Comments on satisfaction with the current LTE program**

| | **Comments** | | |
|---|---|---|---|
| **MCG** | **Positive** | **Neutral/Suggestions** | **Negative** |
| | **Yr 1-2**<br><br>• I feel that being a part of LTE allows me to incorporate a personalized level of medical ethics education into my very busy schedule. The topics of lectures are always very relevant and fascinating and the small group sessions allow me to learn more about ethical dilemmas that I feel are important or controversial.<br>• LTE has helped me to see the value of medical bioethics and the importance it will have throughout my life. LTE has offered me a great outlet to explore my interest in bioethics as well as allowed me to form relationships with mentors who can help me with these ethical dilemmas.<br>• Yes, I do feel that meeting once a month provides great opportunities for discussion on many topics. I do wish that we have more group sessions and interactive class learning opportunities.<br>• I would like a more formal curriculum with a certificate.<br>• I personally do believe that this program is meeting my needs for a formal medical ethics education. I believe that the mixture of case studies, article reviews, and large group presentations has bolstered my | **Yr 1-2**<br><br>• I do feel like I am learning a lot through my participation in the program; however, I do think that there is room for improvement. Through monthly discussions with ethics focused physicians/members of the MCG ethics committee as well as chaplain shadowing, I feel like I have the opportunity to reflect on medical ethics topics in advance to meeting them in a clinical setting. I would like to learn more about the theoretical principals of medical ethics, and I would appreciate more interaction with trained ethicists. I appreciate the practical information learned from ethically focused physicians and chaplains, but I would also like to learn from the perspective of a trained ethicist. Some of the best LTE moments this past year have been with guest speakers who have shared their research and expert knowledge derived from their academic training as well as their active practice in medical ethics at various institutes.<br>• Yes! LTE is an exceptional student-driven program that is continuing to foster my ability to recognize ethical dilemmas and have the confidence to know how to react, what actions to take and how to problem solve a solution. | **Yr 1-2**<br><br>• A more formal curriculum would help greatly in giving a background in bioethics and making sure people are well informed.<br>• It's more discussion/activity based than I hoped. I expected us to learn some philosophical or legal frameworks surrounding the state of current medical ethics. |

| | Comments | | |
|---|---|---|---|
| **MCG** | **Positive** | **Neutral/Suggestions** | **Negative** |
| | judgement making ability and provided me with a framework to work off of as i move forward in my career.<br>• the program is the direct application of how our medical knowledge and clinical knowledge all relate to ethics of a patient. By having in depths conversations with fellow physicians, we understand the compounding factors bioethics plays to the execution of medicine<br>• I am learning more about ethical conversations, practices, and principles through LTE and I am enjoying receiving feedback from my colleagues.<br>• We discuss relevant topics and how hospitals deal with ethical issues. I gain insight into information that will help me become a better physician in the future.<br>• I like the discussion we have concerning ethics the opportunities to shadow. It has broaden my understanding of medical ethics.<br>• Yes, LTE is giving me additional exposure to a variety of experts from MDs who have furthered their education in bioethics to pastoral care to a Phd's point of view.<br>• LTE provides me with the ethical exposure and multiple modes of thought that go into addressing ethical dilemmas<br>• LTE exposes directly to how a hospital deals with ethical issues and through LTE I have access to members of the ethics committee should I have any questions or concerns<br>• We are able to discuss various topics in the realm of ethics in healthcare.<br>• Yes-I enjoy discussing cases with faculty members, and we have the opportunity to begin ethics-related research. | Additionally, the curriculum has given me the opportunity to develop my own personal moral identity and ethical reasoning from the guidance and leadership of many phenomenal clinician, chaplain and lawyer leaders, serving as mentors in our LTE program. We have had the opportunity to learn from the example of many members on the AU Ethics Committee and leaders from our pastoral care team. The only thing that I would like more of is formal discussion of the current literature in bioethics, and I think that a formal certification program would fulfill that missing piece of the curriculum.<br>• While I believe the program does a great job of introducing its members to ethics and how they are handled, it mostly focuses on us listening to doctors or ethicist and simply being exposed to issues. A better approach, I believe, would be having activites that made us think seriously about issues and attempt to find solutions, since that it what we will be doing in our careers.<br>• I appreciate the discussions we get to have with faculty about ethical dilemmas, but I wish we had more opportunities.<br>• I would also like this program to include some didactic instruction and resources in the principles, theories, and academic components of bioethics and medical ethics. | |

| | Comments | | |
|---|---|---|---|
| **MCG** | **Positive** | **Neutral/Suggestions** | **Negative** |
| | **YR 3-4**<br><br>• Having guest lecturers, group-based discussions, pastoral-care shadowing and research opportunities has helped me become more aware of the common medical ethics issues we might see in the hospital as well as made me more comfortable in knowing how to deal with them.<br>• My experience through LTE has provided great experiences highlighting the multi-faceted approach to ethical patient cases. I have enjoyed shadowing pastoral care and sitting in on medical ethics board meetings.<br>• Many of the cases that we discussed during my ethics small group have come up in my rotations and prepared me to think through how I would act.<br>• LTE has provided invaluable experience and exposure to medical ethics.<br>• Opportunities to hear seminars on various topics related to ethics in addition to self learning opportunities have provided a valuable addition to my education.<br>• LTE has done a good job at exposing us to ethical situations in the form of case studies and preparing a lecture on a chosen topic.<br>• I went to a regional campus for M3 and M4 and was, unfortunately, removed from many of the educational experiences available through LTE. While I was more involved, I do think that the exposure to any ethical problem solving was better than what we ever got before.<br>• Provides opportunities to participate in the ethics committee and round with pastoral care teams, as well as small group discussions about ethical topics.<br>• I think a foundational understanding of ethics and how | **YR 3-4**<br><br>• I think the LTE program does a phenomenal job of providing ethical context to the MD program at MCG. I just wish there was a way to provide greater opportunities during the 3rd and 4th year diaspora of students. | **YR 3-4**<br><br>• This is a voluntary study, yet there is no way to opt out to get the study out of our one45 to do's. Please edit the study designs.<br>• rarely meet<br>• I think it would be better to integrate elements of medical ethics into the undergraduate medical education curriculum. For example, as part of a clinical medicine or discipline-based course, there could be lectures utilizing clinical cases to demonstrate how to determine medical capacity, teaching how it is different from competency, and emphasizing what role the physician has in all of this. |

| | Comments | | |
|---|---|---|---|
| **MCG** | **Positive** | **Neutral/Suggestions** | **Negative** |
| | it applies to the practice of medicine is important for any person wishing to engage in human suffering and the tough decisions that come with such an endeavor. | | |

**Table 4a. Importance of ethics to participants' future career, N (%) / Mean ± SD**

| | Program | | | | |
|---|---|---|---|---|---|
| | **CON** (n = 79) | **MCG** (n = 340) | **BS/(D)MD** (n = 70) | **PT** (n = 73) | **Overall** (N = 562) |
| **Q10. How important is an understanding of medical ethics and bioethics principles to your future career in health care?** | | | | | |
| Very important | 58 (73.4%) | 194 (57.1%) | 62 (88.6%) | 36 (49.3%) | 350 (62.3%) |
| Somewhat important | 18 (22.8%) | 115 (33.8%) | 8 (11.4%) | 31 (42.5%) | 172 (30.6%) |
| Neutral | 2 (2.5%) | 21 (6.2%) | 0 (0.0%) | 2 (2.7%) | 25 (4.4%) |
| Somewhat unimportant | 1 (1.3%) | 7 (2.1%) | 0 (0.0%) | 3 (4.1%) | 11 (2.0%) |
| Not important at all | 0 (0.0%) | 3 (0.9%) | 0 (0.0%) | 1 (1.4%) | 4 (0.7%) |
| Mean Score (1: Not Important – 5: Very Important) | 4.68 ± 0.59[a,c] | 4.44 ± 0.78[a,b] | 4.89 ± 0.32[b,d] | 4.34 ± 0.84[c,d] | 4.52 ± 0.74 |

Note: Same letters indicate statistically significant differences between groups

| | Program | | | | | |
|---|---|---|---|---|---|---|
| | **CON** (n = 79) | **MCG[3,4]** (n = 164) | **MCG[1,2]** (n =176) | **BS/(D)MD** (n = 70) | **PT** (n = 73) | **Overall** (N = 562) |
| **Q10.** | | | | | | |
| Very important | 58 (73.4%) | 91 (55.5%) | 103 (58.5%) | 62 (88.6%) | 36 (49.3%) | 350 (62.3%) |
| Somewhat important | 18 (22.8%) | 51 (31.1%) | 64 (36.4%) | 8 (11.4%) | 31 (42.5%) | 172 (30.6%) |
| Neutral | 2 (2.5%) | 15 (9.1%) | 6 (3.4%) | 0 (0.0%) | 2 (2.7%) | 25 (4.4%) |
| Somewhat unimportant | 1 (1.3%) | 4 (2.4%) | 3 (1.7%) | 0 (0.0%) | 3 (4.1%) | 11 (2.0%) |
| Not important at all | 0 (0.0%) | 3 (1.8%) | 0 (0.0%) | 0 (0.0%) | 1 (1.4%) | 4 (0.7%) |
| Mean Score | 4.68 ± 0.59[a] | 4.36 ± 0.89 [a,b] | 4.52 ± 0.65[c] | 4.89 ± 0.32[b-d] | 4.34 ± 0.84[d] | 4.52 ± 0.74 |

Note: Same letters indicate statistically significant differences between groups

**F= 8.747; p<0.0001**

**Table 4b. Please explain your response, if you indicated neutral-not important at all for Q10**

| **Program** | **Comment** |
|---|---|
| **CON** | • As a future anesthetist my job will be to keep the patient alive during the procedure no matter what. Medical ethics is more important in determining wheter the patient should have the procedure in the first place.<br>• Everyone in the medical field (even a medical assistant) needs a medical ethics class!! This should be inbuilt in any and all medical curriculums!<br>• I feel it's a major component of modern nursing.<br>• I think compassion and empathy are more important. Practicing how to fulfill the ally role for the patient rather than learn about the HUGE field of bioethics and how it may pertain to me.<br>• na |
| **MCG** | YR 1-2<br>• I think medical ethics is important to understand right now just to anticipate some of the difficult issues we may face in the hospital during our training and careers. Even though we wouldn't necessarily be |

| Program | Comment |
|---|---|
| | expected to come up with ethical solutions, it would be good not to be blind sided by common ethical dilemmas and to be able to handle them the best we can in the moment that they occur, in case there are no superiors around to guide our judgement. I would feel more comfortable being alone with patients if I had an understanding of basic ethical issues. Bioethics is a little different, kind of advanced for this level, but good to think about for people who are interested.<br>• It is important to act ethically, but in depth knowledge about medical ethics and various school of thoughts is not necessary to do so.<br>• I think that people have difering opinions about ethics, and that as long as you adhere to your own ethics and try your best not to harm anyone, you will be able to figure it out<br>• I chose somewhat important because I think looking at all the things a job in the medical field has to do, medical ethics probably may not be the most important thing. I think most people who are going into the field want to help people and will probably already act ethically as an inherent characteristic.<br>• If I encountered a medical ethical dilemma, I would approach colleagues/ethics council to make sure all sides are fairly represented, I am acting within the law and within my institution's (if applicable) rules.<br><br>YR 3-4<br><br>• I am going into a specialty that will not have as much patient contact.<br>• This is a voluntary study, yet there is no way to opt out to get the study out of our one45 to do's. Please edit the study designs. |
| **BS/(D)MD** | • No Comment |
| **PT** | • n/a (3)<br>• A certificate, if it could be grafted in to the program would be interesting.<br>• Good to know, but not heavily emphesized because not conducting research on humans or the environment.<br>• I don't even know what bioehtics is.<br>• I don't fully understand what bioethics are.<br>• In physical therapy, we are not responsible for "pulling the plug" so to say, but still need to know how to respect the decisions & choices of our patients<br>• It is important to know what can and cannot be done when working so closely and intimately with patients on a daily basis.<br>• It sounds good to know about.<br>• Need to know the basics |

**Table 5. Interest in pursuing a medical ethics/bioethics graduate certificate or master's program, N (%) / Mean ± SD**

| | Program | | | | |
|---|---|---|---|---|---|
| | **CON** (n = 79) | **MCG** (n = 340) | **BS/(D)MD** (n = 70) | **PT** (n = 73) | **Overall** (N = 562) |
| **Q11. Please rate your level of interest in pursuing a medical ethics and bioethics graduate certificate or master's program** | | | | | |
| Very likely | 5 (6.3%) | 32 (9.4%) | 6 (8.6%) | 1 (1.4%) | 44 (7.8%) |
| Somewhat likely | 18 (22.8%) | 67 (19.7%) | 23 (32.9%) | 9 (12.3%) | 117 (20.8%) |
| Neutral | 19 (24.1%) | 82 (24.1%) | 23 (32.9%) | 20 (27.4%) | 144 (25.6%) |
| Somewhat unlikely | 14 (17.7%) | 72 (21.2%) | 14 (20.0%) | 17 (23.3%) | 117 (20.8%) |
| Very unlikely | 23 (29.1%) | 87 (25.6%) | 4 (5.7%) | 26 (35.6%) | 140 (24.9%) |
| Mean Score (1: Very Unlikely – 5: Very Likely) | 2.59 ± 1.30[a] | 2.66 ± 1.30[b,c] | 3.19 ± 1.04[a,b,d] | 2.21 ± 1.11[c,d] | 2.66 ± 1.27 |

Note: Same letters indicate statistically significant differences between groups

**$F = 5.214; p < 0.0001$**

| | Program | | | | | |
|---|---|---|---|---|---|---|
| | **CON** (n = 79) | **MCG[3,4]** (n = 164) | **MCG[1,2]** (n =176) | **BS/(D)MD** (n = 70) | **PT** (n = 73) | **Overall** (N = 562) |
| **Q11.** | | | | | | |
| Very likely + Somewhat likely | 23 (29.1%) | 37 (22.6%) | 62 (35.2%) | 29 (41.4%) | 10 (13.7%) | 161 (28.6%) |
| Neutral + Somewhat unlikely + Very unlikely | 56 (70.9%) | 127 (77.4%) | 114 (64.8%) | 41 (58.6%) | 63 (86.3%) | 401 (71.4%) |

**$\chi^2 = 20.283; p < 0.0001$**

***Note:*** Only those that responded "Somewhat likely" or "Very likely" for Q11 responded to Q12-15

**Table 6. Motivational factors for interest in ethics certificate/program, N(%)**

| | Program | | | | |
|---|---|---|---|---|---|
| | **CON** (n = 23) | **MCG** (n = 99) | **BS/(D)MD** (n = 29) | **PT** (n = 10) | **Overall** (N = 161) |
| **Q12. Which of the following factors are part of your motivation?** | | | | | |
| Importance to career | 18 (78.3%) | 81 (81.8%) | 25 (86.2%) | 6 (60.0%) | 130 (80.7%) |
| Desire to help others | 14 (60.9%) | 78 (78.8%) | 24 (82.8%) | 8 (80.0%) | 124 (77.0%) |
| Enjoyment of learning | 13 (56.5%) | 67 (67.7%) | 20 (69.0%) | 4 (40.0%) | 104 (64.6%) |
| Desire to provide healthcare ethics consultation | 14 (60.9%) | 52 (52.5%) | 9 (31.0%) | 6 (60.0%) | 81 (50.3%) |
| Advancement in residency application | 1 (4.3%) | 50 (50.5%) | 17 (58.6%) | 2 (20.0%) | 70 (43.5%) |
| Research opportunities | 7 (30.4%) | 18 (18.2%) | 10 (34.5%) | 1 (10.0%) | 36 (22.4%) |
| Desire to publish bioethics research | 4 (17.4%) | 19 (19.2%) | 3 (10.3%) | 0 (0.0%) | 26 (16.1%) |
| Desire to teach bioethics | 7 (30.4%) | 15 (15.2%) | 3 (10.3%) | 1 (10.0%) | 26 (16.1%) |
| Other | 3 (13.0%) | 3 (3.0%) | 0 (0.0%) | 0 (0.0%) | 6 (4.7%) |

| | Program | | | | | |
|---|---|---|---|---|---|---|
| | **CON** (n = 23) | **MCG[3,4]** (n = 37) | **MCG[1,2]** (n =62) | **BS/(D)MD** (n = 29) | **PT** (n = 10) | **Overall** (N = 161) |
| **Q12.** | | | | | | |
| Importance to career | 18 (78.3%) | 30 (81.1%) | 51 (82.3%) | 25 (86.2%) | 6 (60.0%) | 130 (80.7%) |
| Desire to help others | 14 (60.9%) | 30 (81.1%) | 48 (77.4%) | 24 (82.8%) | 8 (80.0%) | 124 (77.0%) |
| Enjoyment of learning | 13 (56.5%) | 27 (73.0%) | 40 (64.5%) | 20 (69.0%) | 4 (40.0%) | 104 (64.6%) |
| Desire to provide healthcare ethics consultation | 14 (60.9%) | 21 (56.8%) | 31 (50.0%) | 9 (31.0%) | 6 (60.0%) | 81 (50.3%) |
| Advancement in residency application | 1 (4.3%) | 16 (43.2%) | 34 (54.8%) | 17 (58.6%) | 2 (20.0%) | 70 (43.5%) |
| Research opportunities | 7 (30.4%) | 6 (16.2%) | 12 (19.4%) | 10 (34.5%) | 1 (10.0%) | 36 (22.4%) |
| Desire to publish bioethics research | 4 (17.4%) | 7 (18.9%) | 12 (19.4%) | 3 (10.3%) | 0 (0.0%) | 26 (16.1%) |
| Desire to teach bioethics | 7 (30.4%) | 7 (18.9%) | 8 (12.9%) | 3 (10.3%) | 1 (10.0%) | 26 (16.1%) |
| Other | 3 (13.0%) | 1 (2.7%) | 2 (3.2%) | 0 (0.0%) | 0 (0.0%) | 6 (4.7%) |

*Other (specify):*

- I think it's really important for people making decisions to understand what is ethical whether that's what a book says or not. Sometimes ethics isn't a black or white issue. Also we as people in medicine need to have an idea of what laws surround our decisions about ethics.
- I'd like to have as much experience under my belt as possible when it comes to being a well prepared and well rounded healthcare practitioner.
- It would give me more knowledge needed to speak like a lawyer (which is important when discussing about hot button issues) regarding end of life care.
- Leadership position.
- I would not pursue a master's in medical ethics but would consider a certification program. I would like to receive more of this education but do not want it to interfere with my other studies but rather want it to enhance my other studies in medicine.

- Personal moral judgement & ethical reasoning
- Having a better understanding of ethical situations and how to proceed in those situations.
- Protect vulnerable populations

**Table 7a. Program of interest, N (%) / Mean ± SD**

| | Program | | | | |
|---|---|---|---|---|---|
| | **CON** (n = 23) | **MCG** (n = 99) | **BS/(D)MD** (n = 29) | **PT** (n = 10) | **Overall** (N = 161) |
| **Q13. Which program design are you most likely to be interested in?** | | | | | |
| ***Graduate Certificate*** | | *missing (n=5)* | | | |
| Very likely | 18 (78.3%) | 49 (52.1%) | 14 (48.3%) | 2 (20.0%) | 83 (53.2%) |
| Somewhat likely | 3 (13.0%) | 41 (43.6%) | 10 (34.5%) | 4 (40.0%) | 58 (37.2%) |
| Neutral | 2 (8.7%) | 4 (4.3%) | 4 (13.8%) | 2 (20.0%) | 12 (7.7%) |
| Somewhat unlikely | 0 (0.0%) | 0 (0.0%) | 1 (3.4%) | 1 (10.0%) | 2 (1.3%) |
| Very unlikely | 0 (0.0%) | 0 (0.0%) | 0 (0.0%) | 1 (10.0%) | 1 (0.6%) |
| Mean Score | 4.70 ± 0.64[a] | 4.48 ± 0.58[b] | 4.28 ± 0.84[c] | 3.50 ± 1.27[a-c] | 4.41 ± 0.74 |
| **$F = 6.002$ ; $p < 0.0001$** | | | | | |
| ***Master's degree*** | | *missing (n=3)* | | | |
| Very likely | 2 (8.7%) | 6 (6.3%) | 3 (10.3%) | 1 (10.0%) | 12 (7.6%) |
| Somewhat likely | 7 (30.4%) | 23 (24.0%) | 6 (20.7%) | 1 (10.0%) | 37 (23.4%) |
| Neutral | 3 (13.0%) | 25 (26.0%) | 12 (41.4%) | 6 (60.0%) | 46 (29.1%) |
| Somewhat unlikely | 7 (30.4%) | 34 (35.4%) | 3 (10.3%) | 1 (10.0%) | 45 (28.5%) |
| Very unlikely | 4 (17.4%) | 8 (8.3%) | 5 (17.2%) | 1 (10.0%) | 18 (11.4%) |
| Mean Score | 2.83 ± 1.30 | 2.84 ± 1.08 | 2.97 ± 1.21 | 3.00 ± 1.05 | 2.87 ± 1.13 |
| **$F = 0.256$ ; $p = 0.906$** | | | | | |

*Note: Mean Score (1: Very Unlikely – 5: Very Likely)*

| | Program | | | | | |
|---|---|---|---|---|---|---|
| | **CON** (n = 23) | **MCG[3,4]** (n = 37) | **MCG[1,2]** (n =59) | **BS/(D)MD** (n = 29) | **PT** (n = 10) | **Overall** (N = 161) |
| **Q13.** | | | | | | |
| ***Graduate Certificate*** | | *missing (n=1)* | *missing (n=1)* | | | N=156 |
| Very likely + Somewhat likely | 21 (91.3%) | 35 (97.2%) | 55 (94.8%) | 24 (82.8%) | 6 (60.0%) | 141 (90.4%) |
| Neutral + Somewhat unlikely +Very unlikely | 2 (8.7%) | 1 (2.8%) | 3 (5.2%) | 5 (17.2%) | 4 (40.0%) | 15 (9.6%) |
| **$\chi^2 = 15.840$ ; $p = 0.003$** | | | | | | |
| ***Master's degree*** | | | *missing (n=3)* | | | N=158 |
| Very likely Somewhat likely | 9 (39.1%) | 10 (27.0%) | 19 (32.2%) | 9 (31.0%) | 2 (20.0%) | 49 (31.0%) |
| Neutral + Somewhat unlikely + Very unlikely | 14 (60.9%) | 27 (73.0%) | 40 (67.8%) | 20 (69.0%) | 8 (80.0%) | 109 (69.0%) |
| **$\chi^2 = 1.589$ ; $p = 0.811$** | | | | | | |

**Table 7b. Program format design of interest, N (%) / Mean ± SD**

| | Program | | | | |
|---|---|---|---|---|---|
| | **CON** (n = 23) | **MCG** (n = 99) | **BS/(D)MD** (n = 29) | **PT** (n = 10) | **Overall** (N = 161) |
| **Q14. How likely are you to be interested in the following program format designs?** | | | | | |
| ***Fully in class program*** | | | | | |
| Very likely | 0 (0.0%) | 13 (14.0%) | 8 (27.6%) | 2 (20.0%) | 23 (14.8%) |
| Somewhat likely | 3 (13.0%) | 38 (40.9%) | 9 (31.0%) | 0 (0.0%) | 50 (32.3%) |
| Neutral | 3 (13.0%) | 24 (25.8%) | 9 (31.0%) | 2 (20.0%) | 38 (24.5%) |
| Somewhat unlikely | 9 (39.1%) | 11 (11.8%) | 3 (10.3%) | 5 (50.0%) | 28 (18.1%) |
| Very unlikely | 8 (34.8%) | 7 (7.5%) | 0 (0.0%) | 1 (10.0%) | 16 (10.3%) |
| Mean Score | 2.04 ± 1.02[a,b] | 3.42 ± 1.11[a] | 3.76 ± 0.99[b] | 2.70 ± 1.34 | 3.23 ± 1.21 |
| ***Fully online program*** | | | | | |
| Very likely | 14 (60.9%) | 26 (28.3%) | 6 (20.7%) | 1 (10.0%) | 47 (30.5%) |
| Somewhat likely | 4 (17.4%) | 34 (37.0%) | 11 (37.9%) | 8 (80.0%) | 57 (37.0%) |
| Neutral | 2 (8.7%) | 10 (10.9%) | 7 (24.1%) | 0 (0.0%) | 19 (12.3%) |
| Somewhat unlikely | 2 (8.7%) | 10 (10.9%) | 3 (10.3%) | 0 (0.0%) | 15 (9.7%) |
| Very unlikely | 1 (4.3%) | 12 (13.0%) | 2 (6.9%) | 1 (10.0%) | 16 (10.4%) |
| Mean Score | 4.22 ± 1.20 | 3.57 ± 1.35 | 3.55 ± 1.15 | 3.80 ± 1.03 | 3.68 ± 1.29 |
| ***Hybrid format (50/50)*** | | | | | |
| Very likely | 8 (34.8%) | 34 (36.6%) | 8 (27.6%) | 4 (40.0%) | 54 (34.8%) |
| Somewhat likely | 7 (30.4%) | 47 (50.5%) | 11 (37.9%) | 3 (30.0%) | 68 (43.9%) |
| Neutral | 3 (13.0%) | 8 (8.6%) | 7 (24.1%) | 3 (30.0%) | 21 (13.5%) |
| Somewhat unlikely | 3 (13.0%) | 3 (3.2%) | 3 (10.3%) | 0 (0.0%) | 9 (5.8%) |
| Very unlikely | 2 (8.7%) | 1 (1.1%) | 0 (0.0%) | 0 (0.0%) | 3 (1.9%) |
| Mean Score | 3.70 ± 1.33 | 4.18 ± 0.81 | 3.83 ± 0.97 | 4.10 ± 0.88 | 4.04 ± 0.95 |

*Note: Mean Score (1: Very Unlikely – 5: Very Likely)*

| | Program | | | | | |
|---|---|---|---|---|---|---|
| | **CON** (n = 23) | **MCG[3,4]** (n = 37) | **MCG[1,2]** (n =62) | **BS/(D)MD** (n = 29) | **PT** (n = 10) | **Overall** (N = 161) |
| **Q14.** | | | | | | |
| ***Fully in class program*** | | | | | | |
| Very likely | 0 (0.0%) | 3 (8.1%) | 10 (17.9%) | 8 (27.6%) | 2 (20.0%) | 23 (14.8%) |
| Somewhat likely | 3 (13.0%) | 18 (48.6%) | 20 (35.7%) | 9 (31.0%) | 0 (0.0%) | 50 (32.3%) |
| Neutral | 3 (13.0%) | 8 (21.6%) | 16 (28.6%) | 9 (31.0%) | 2 (20.0%) | 38 (24.5%) |
| Somewhat unlikely | 9 (39.1%) | 5 (13.5%) | 6 (10.7%) | 3 (10.3%) | 5 (50.0%) | 28 (18.1%) |
| Very unlikely | 8 (34.8%) | 3 (8.1%) | 4 (7.1%) | 0 (0.0%) | 1 (10.0%) | 16 (10.3%) |
| Mean Score | 2.04 ± 1.02[a-c] | 3.35 ± 1.09[a] | 3.46 ± 1.13[b] | 3.76 ± 0.99[c] | 2.70 ± 1.34 | 3.23 ± 1.21 |
| ***Fully online program*** | | | | | | |
| Very likely | 14 (60.9%) | 11 (30.6%) | 15 (26.8%) | 6 (20.7%) | 1 (10.0%) | 47 (30.5%) |
| Somewhat likely | 4 (17.4%) | 14 (38.9%) | 20 (35.7%) | 11 (37.9%) | 8 (80.0%) | 57 (37.0%) |
| Neutral | 2 (8.7%) | 4 (11.1%) | 6 (10.7%) | 7 (24.1%) | 0 (0.0%) | 19 (12.3%) |
| Somewhat unlikely | 2 (8.7%) | 3 (8.3%) | 7 (12.5%) | 3 (10.3%) | 0 (0.0%) | 15 (9.7%) |

| | | | | | | |
|---|---|---|---|---|---|---|
| Very unlikely | 1 (4.3%) | 4 (11.1%) | 8 (14.3%) | 2 (6.9%) | 1 (10.0%) | 16 (10.4%) |
| Mean Score | 4.22 ± 1.20 | 3.69 ± 1.30 | 3.48 ± 1.39 | 3.55 ± 1.15 | 3.80 ± 1.03 | 3.68 ± 1.29 |
| ***Hybrid format (50/50)*** | | | | | | |
| Very likely | 8 (34.8%) | 13 (35.1%) | 21 (37.5%) | 8 (27.6%) | 4 (40.0%) | 54 (34.8%) |
| Somewhat likely | 7 (30.4%) | 21 (56.8%) | 26 (46.4%) | 11 (37.9%) | 3 (30.0%) | 68 (43.9%) |
| Neutral | 3 (13.0%) | 2 (5.4%) | 6 (10.7%) | 7 (24.1%) | 3 (30.0%) | 21 (13.5%) |
| Somewhat unlikely | 3 (13.0%) | 1 (2.7%) | 2 (3.6%) | 3 (10.3%) | 0 (0.0%) | 9 (5.8%) |
| Very unlikely | 2 (8.7%) | 0 (0.0%) | 1 (1.8%) | 0 (0.0%) | 0 (0.0%) | 3 (1.9%) |
| Mean Score | 3.70 ± 1.33 | 4.24 ± 0.68 | 4.14 ± 0.88 | 3.83 ± 0.97 | 4.10 ± 0.88 | 4.04 ± 0.95 |

*Other comments (only included for MCG survey):*

- The best aspects of LTE are the group discussions and in-person engagement activities. I cannot imagine the course taught fully online. Engaged reflection on these important topics is much more challenging when done in solitude because there is no opportunity for reflection on opposing view points. However, I do understand the utility of teaching some of the principles of medical ethics or history of medical ethics online.
- Include practical experience (e.g. ethics committee attendance), research opportunities, and case discussion.
- I would much prefer the master's, but I would not consider it if it added an additional year to my studies (unless that year could be completed after residency). A self-paced program would be a better option. I would not delay residency for it.

**Table 8. Educational components, N(%)**

| | Program | | | | |
|---|---|---|---|---|---|
| | **CON** (n = 23) | **MCG** (n = 99) | **BS/(D)MD** (n = 29) | **PT** (n = 10) | **Overall** (N = 161) |
| **Q15. Which of the following educational components of a potential medical ethics and bioethics curriculum are you interested in?** | | | | | |
| Ethics case-based discussions | 17 (73.9%) | 82 (82.8%) | 25 (86.2%) | 6 (60.0%) | 130 (80.7%) |
| Ethics guest lectures | 19 (82.6%) | 76 (76.8%) | 24 (82.8%) | 7 (70.0%) | 126 (78.3%) |
| Ethics discussion in small peer groups | 15 (65.2%) | 65 (65.7%) | 17 (58.6%) | 4 (40.0%) | 101 (62.7%) |
| Faculty-student mentorship sessions | 14 (60.9%) | 72 (72.7%) | 10 (34.5%) | 3 (30.0%) | 99 (61.5%) |
| Introductory course in foundations of bioethics | 12 (52.2%) | 62 (62.6%) | 20 (69.0%) | 6 (60.0%) | 100 (62.1%) |
| Palliative care/hospice clerkship rotation | 14 (60.9%) | 61 (61.6%) | 15 (51.7%) | 4 (40.0%) | 94 (58.4%) |
| AU Health ethics consultation review committee | 11 (47.8%) | 64 (64.6%) | 9 (31.0%) | 5 (50.0%) | 89 (55.3%) |
| Reading seminar on landmark medical ethics cases | 12 (52.2%) | 54 (54.5%) | 10 (34.5%) | 3 (30.0%) | 79 (49.1%) |
| Pastoral care shadowing | 4 (17.4%) | 55 (55.6%) | 11 (37.9%) | 3 (30.0%) | 73 (45.3%) |
| Research/publication opportunities | 8 (34.8%) | 45 (45.5%) | 13 (44.8%) | 2 (20.0%) | 68 (42.2%) |
| Student ethics committee | 7 (30.4%) | 46 (46.5%) | 7 (24.1%) | 3 (30.0%) | 63 (39.1%) |
| Other | 2 (8.7%) | 0 (0.0%) | 0 (0.0%) | 0 (0.0%) | 2 (1.2%) |

| | Program | | | | | |
|---|---|---|---|---|---|---|
| | **CON** (n = 23) | **MCG**[3,4] (n = 37) | **MCG**[1,2] (n =62) | **BS/(D)MD** (n = 29) | **PT** (n = 10) | **Overall** (N = 161) |
| **Q15.** | | | | | | |
| Ethics case-based discussions | 17 (73.9%) | 33 (89.2%) | 49 (79.0%) | 25 (86.2%) | 6 (60.0%) | 130 (80.7%) |
| Ethics guest lectures | 19 (82.6%) | 30 (81.1%) | 46 (74.2%) | 24 (82.8%) | 7 (70.0%) | 126 (78.3%) |
| Ethics discussion in small peer groups | 15 (65.2%) | 21 (56.8%) | 44 (71.0%) | 17 (58.6%) | 4 (40.0%) | 101 (62.7%) |
| Faculty-student mentorship sessions | 14 (60.9%) | 27 (73.0%) | 45 (72.6%) | 10 (34.5%) | 3 (30.0%) | 99 (61.5%) |
| Introductory course in foundations of bioethics | 12 (52.2%) | 26 (70.3%) | 36 (58.1%) | 20 (69.0%) | 6 (60.0%) | 100 (62.1%) |
| Palliative care/hospice clerkship rotation | 14 (60.9%) | 26 (70.3%) | 35 (56.5%) | 15 (51.7%) | 4 (40.0%) | 94 (58.4%) |
| AU Health ethics consultation review committee | 11 (47.8%) | 26 (70.3%) | 38 (61.3%) | 9 (31.0%) | 5 (50.0%) | 89 (55.3%) |
| Reading seminar on landmark medical ethics cases | 12 (52.2%) | 22 (59.5%) | 32 (51.6%) | 10 (34.5%) | 3 (30.0%) | 79 (49.1%) |
| Pastoral care shadowing | 4 (17.4%) | 21 (56.8%) | 34 (54.8%) | 11 (37.9%) | 3 (30.0%) | 73 (45.3%) |
| Research/publication opportunities | 8 (34.8%) | 17 (45.9%) | 28 (45.2%) | 13 (44.8%) | 2 (20.0%) | 68 (42.2%) |
| Student ethics committee | 7 (30.4%) | 21 (56.8%) | 25 (40.3%) | 7 (24.1%) | 3 (30.0%) | 63 (39.1%) |
| Other | 2 (8.7%) | 0 (0.0%) | 0 (0.0%) | 0 (0.0%) | 0 (0.0%) | 2 (1.2%) |

*Other (specify):*

- Identification of and action on relevant community ethical issues, in particular vis a vis reproductive justice

- Would be very interested in landmark medical ethics cases
